# The genus *Micromonospora* as a model microorganism for bioactive natural product discovery†

**DOI:** 10.1039/d0ra04025h

**Published:** 2020-06-08

**Authors:** Mohamed S. Hifnawy, Mohamed M. Fouda, Ahmed M. Sayed, Rabab Mohammed, Hossam M. Hassan, Sameh F. AbouZid, Mostafa E. Rateb, Alexander Keller, Martina Adamek, Nadine Ziemert, Usama Ramadan Abdelmohsen

**Affiliations:** Department of Pharmacognosy, Faculty of Pharmacy, Cairo University Cairo Egypt 11787; Department of Pharmacognosy, Faculty of Pharmacy, Nahda University Beni-Suef Egypt 62513; Department of Pharmacognosy, Faculty of Pharmacy, Beni-Suef University Beni-Suef Egypt 62514; School of Computing, Engineering and Physical Sciences, University of the West of Scotland Paisley PA1 2BE UK; Center for Computational and Theoretical Biology, Biocenter, University of Würzburg Hubland Nord 97074 Würzburg Germany; Interfaculty Institute of Microbiology and Infection Medicine Tübingen, University of Tübingen Tübingen Germany nadine.ziemert@uni-tuebingen.de; German Centre for Infection Research (DZIF) Partner Site Tübingen Tübingen Germany; Department of Pharmacognosy, Faculty of Pharmacy, Minia University 61519 Minia Egypt usama.ramadan@mu.edu.eg; Department of Pharmacognosy, Faculty of Pharmacy, Deraya University, Universities Zone P.O. Box 61111 New Minia City 61519 Minia Egypt

## Abstract

This review covers the development of the genus *Micromonospora* as a model for natural product research and the timeline of discovery progress from the classical bioassay-guided approaches through the application of genome mining and genetic engineering techniques that target specific products. It focuses on the reported chemical structures along with their biological activities and the synthetic and biosynthetic studies they have inspired. This survey summarizes the extraordinary biosynthetic diversity that can emerge from a widely distributed actinomycete genus and supports future efforts to explore under-explored species in the search for novel natural products.

## Abstract 2

We explore the genus *Micromonospora* as a model for natural product research and the discovery progress from the classical bioassay-guided approaches through to the application of genome mining and genetic engineering techniques that target specific products.

## Graphical abstract

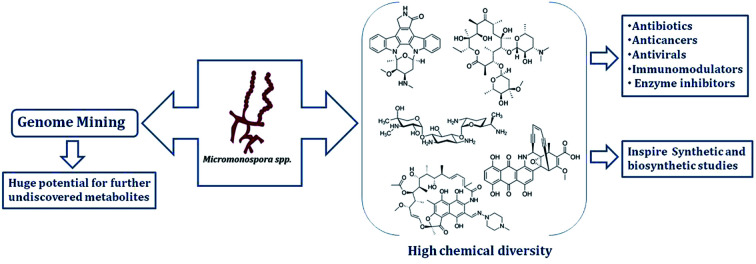

## 1. Introduction

Microbial natural products are considered an essential component of today's drug arsenal. They revolutionized medicine in the 20th century not just by treating mortal diseases, but also by enabling life-saving procedures such as heart transplantation or catheterization to be performed.^1^ The interest in this historical drug resource is decreasing due to the continued rediscovery of known metabolites. However, urgent demand for new drug leads to combat antibiotic-resistant pathogenic infections and other life-threatening diseases, together with the low returns from alternative discovery platforms, has led to the revival of natural product research, especially from microbial sources.^2^

**Fig. 1:**
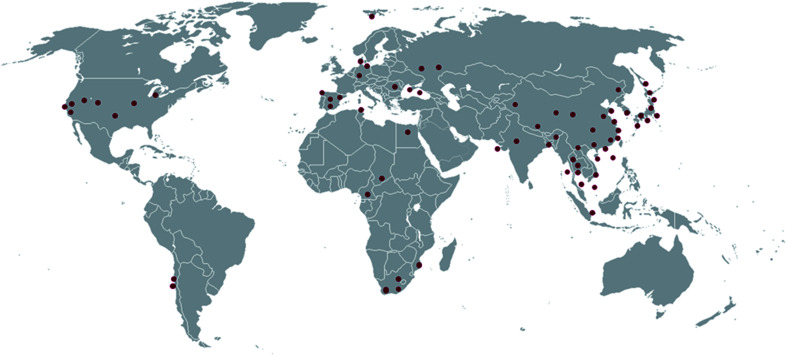
Global locations from which the genus *Micromonospora* has been reported.

**Fig. 2:**
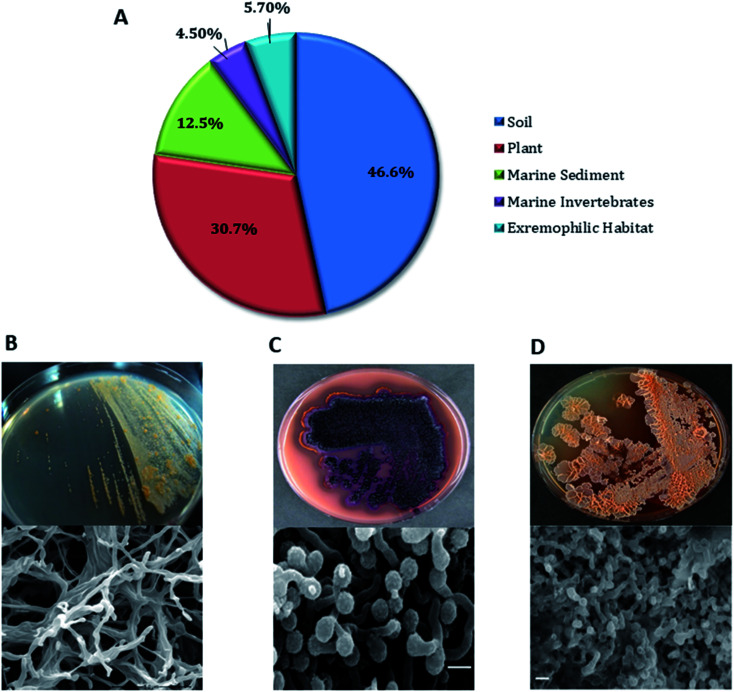
(A) Percentage distribution of *Micromonospora* species by habitats. (B) *M. rifamycinica*,^14^ the main producer of rifamycin antibiotic (177), and it always reported from marine habitats. (C) *M. echinospora*,^15^ the main producer of gentamicin antibiotics (85–87). (D) *M. rosaria*,^15^ the main producer of rosamicin antibiotic (7).

**Fig. 3:**
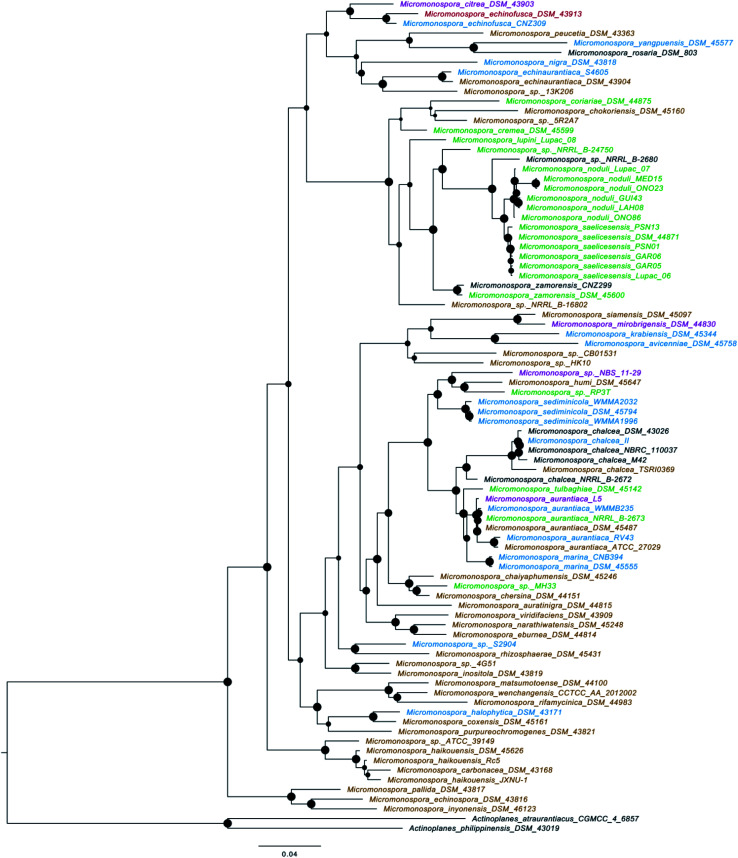
Phylogenomic tree of all *Micromonospora* genomes available at EzBioCloud with *Actinoplanes* as the outgroup. The tree was calculated using bcgTree. Node bubbles represent maximum likelihood bootstrap support values with 1000 replicates. The colors of names are representative for environmental origin of the isolate associated with the genome, with marine: blue, freshwater: purple, brown: soil, animal (feces): red, plant associated (mostly roots): green and black: origin unknown.

*Actinobacteria* represent one of the largest bacterial phyla which widespread across all natural habitats and clearly represented in both terrestrial and marine ecosystems^3,4^ as well as extreme environments.^5^ Approximately 70% of the naturally-derived compounds that are currently in the market or under clinical trials are derived from them.^6,7^ The genus *Micromonospora*, a member of family *Micromonosporaceae* was originally proposed by Ørskov in 1923. Members of this genus are Gram-positive, spore-forming aerobic *Actinobacteria* that own unique morphological characteristics such as single spore attached to short substrate hyphae. Also, they possess carotenoid mycelial pigments that showing yellow, orange, red, purple, brown or black colonies.^8^ Species belonging to this genus are widespread across diverse geographical habitats *viz*; soil, mangrove sediment, marine sediment, plants, and extreme habitats (*e.g.* hyper-arid deserts, deep-sea sediments and hypersaline lakes) (Fig. 1 and 2).^9^ To date, this genus comprised 88 genuinely named species (http://www.bacterio.net) with about 83% of them are of terrestrial origin including plants endophytes and extreme habitats-derived-strains. On the other hand, the marine-derived species are frequently reported from both marine sediment and sponges (Fig. 2). A phylogenomic tree (Fig. 3) of all *Micromonospora* genomes available at EzBioCloud was calculated using bcgTree and 1000 bootstrap replicates. Based on amino-acid sequences of whole-genome data, this algorithm identified 107 crucial single-copy genes and performs a partitioned maximum-likelihood analysis with RaxML. The phylogenomic tree revealed that the genus *Micromonospora* inhabits a wide range of habitats, with environmental origin not associated with monophyletic clades. This indicates broad adaptability of the whole genus to inhabit a variety of environments.

*Micromonospora* has been regarded as a reservoir of antimicrobial agents and other bioactive metabolites^10^ with a biosynthetic potential comparable to the genus *Streptomyces*.^11^ There are two previous reviews that briefly discussed the antibacterial metabolites produced by members of this genus.^12,13^

The main aim of this review is to emphasize the development of the *Micromonospora* genus as a model for microbial natural product research, focusing on the discovery of its novel natural products along with their important biological activities in a chronological manner. Many uncommon *Micromonospora*-derived metabolites that have inspired synthetic, biosynthetic, and mechanistic scientists are highlighted. Early discovery efforts utilized the traditional bioassay-guided approaches while some of the recent reports result from the application of genetic engineering and metabolomics approaches. Additionally, this review summarizes the biosynthetic capacities of *Micromonospora* illustrating how microbial natural products is coupled with biological and biochemical investigations in an interdisciplinary manner for the discovery of new natural drug leads.

## 2. *Micromonospora*: a promising genus to mine for secondary metabolites

**Fig. 4:**
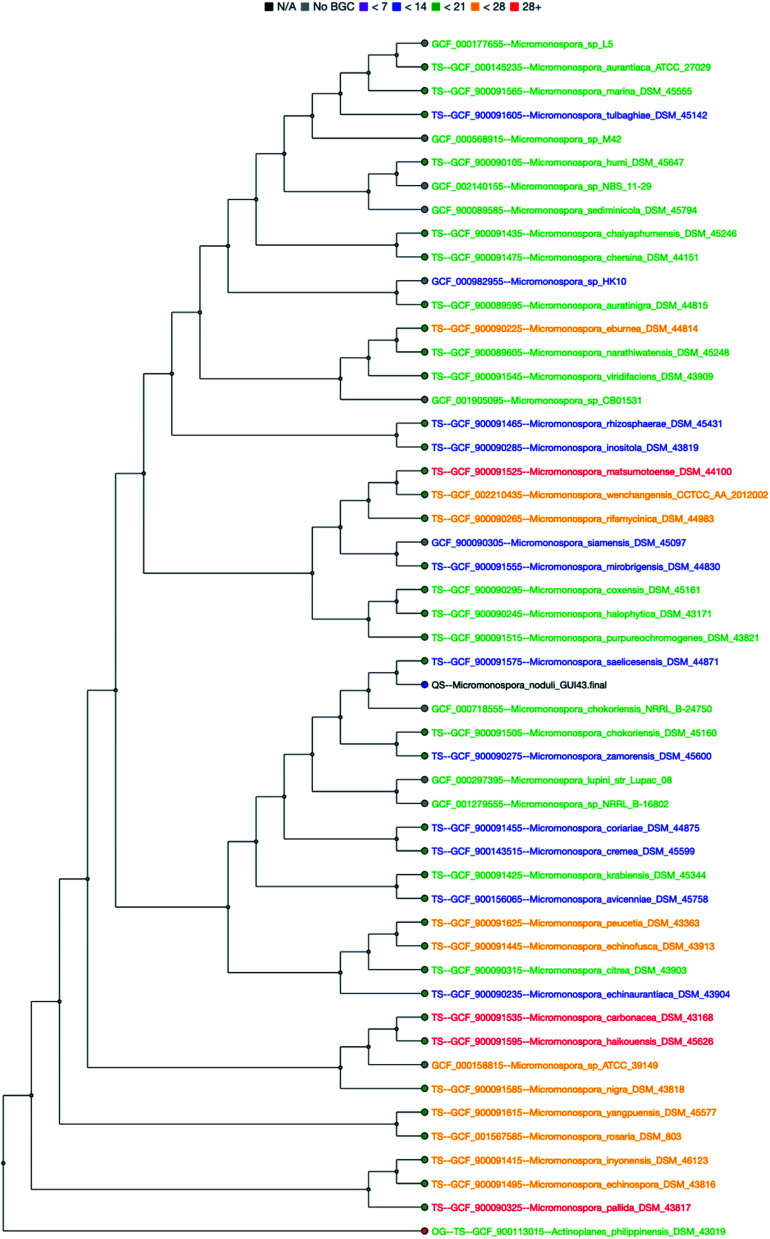
*Micromonospora* phylogenetic tree generated with autoMLST. Color code is representing the number of BGCs identified by antiSMASH.

To evaluate the potential of the genus *Micromonospora* to produce secondary metabolites, we used a combination of bioinformatics tools for analyzing the distribution of the relationships between biosynthetic gene clusters (BGCs). autoMLST, a tool for phylogenomic classification of bacterial genomes, contains a database of bacterial genomes and their biosynthetic potential that is precalculated from antiSMASH results.^16,17^ Using *M. noduli* GUI 43 as a query sequence, a phylogenetic tree was generated from 50 *Micromonospora* strains and representative species (Fig. 4). The biosynthetic potential (number of BGCs detected by antiSMASH) of each *Micromonospora* strain is reflected in the color code. Of the 50 analyzed *Micromonospora* strains, 22 had between 7–13 BGCs (blue), 12 strains had between 14–20 BGCs (green), 11 strains had between 21–27 BGCs (yellow) and four strains showed even more than 28 BGCs (red). This shows that *Micromonospora* has a high potential for producing secondary metabolites.

For further analysis, a database of 87 genomes retrieved from EzBioCloud was submitted to antiSMASH (for genomes also see Fig. 4). Based on the antiSMASH results, we furthermore characterized the distribution of BGCs from *Micromonospora* using network analyses with BigScape.^18^ Overall, it was possible to identify 2387 BGCs that could be grouped into 1033 BGC-families (Table 1 and Fig. S1–S8†). Of these families, 773 were singletons, meaning that they are only found in a single strain. Thereby the majority of BGC-families belong to the type 1 polyketide synthases (T1PKS) and the non-ribosomal peptide synthetases (NRPS). To identify which of the *Micromonospora* BGC families is already encoding a known compound, the dataset from the MIBiG database was added to the network analysis.^19^ Only 15 of 1033 BGCs belonged to or showed a high similarity to the already known BGCs, most of which were polyketides. This not only confirms that the genus *Micromonospora* has a huge potential for producing secondary metabolites, but also that there are potentially a lot of novel metabolites to discover.

**Table 1:** Biosynthetic gene clusters and gene cluster families from 87 *Micromonospora* genomes

Cluster type	BGCs	Singletons	Families	Known (MIBiG)
PKSI	437	187	240	5
Others	328	119	158	2
Saccharides	47	10	18	1
Other PKS	280	56	79	5
PKS–NRP hybrids	174	71	92	0
NRPS	428	180	240	1
Terpene	403	40	68	1
RiPPs	290	110	138	0
Total	2387	773	1033	15

## 3. *Micromonospora*-derived natural products

### 3.1. Macrolides

**Fig. 5:**
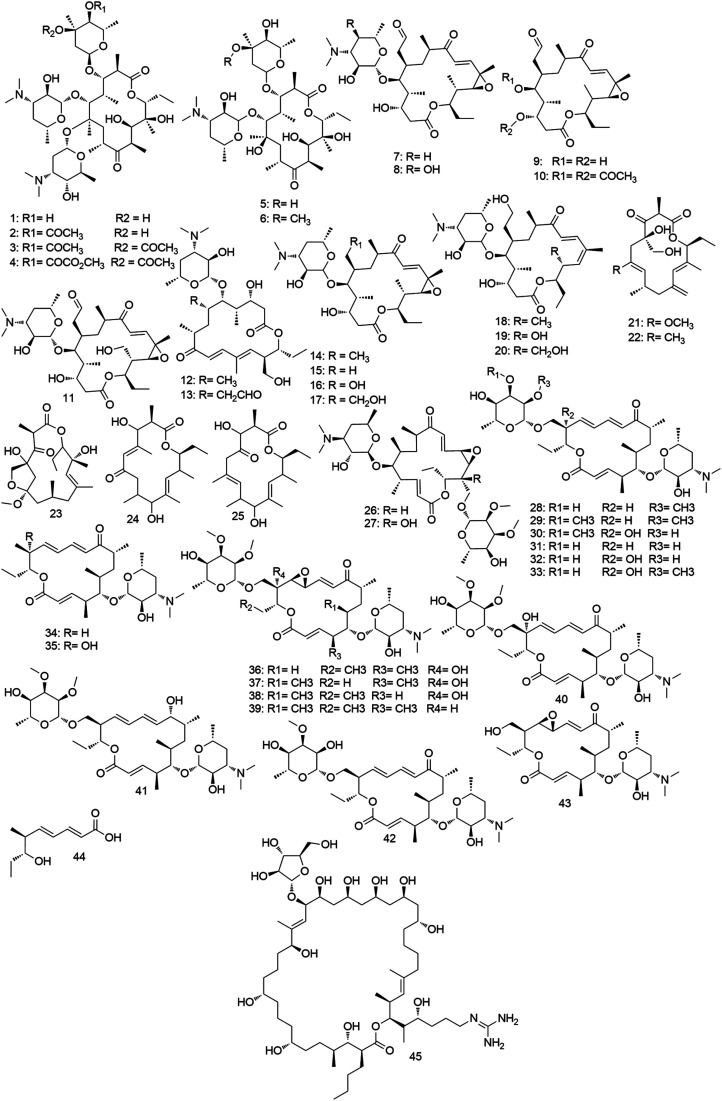
Macrolides derived from *Micromonospora* with antimicrobial activities (compounds 1–45).

**Fig. 6:**
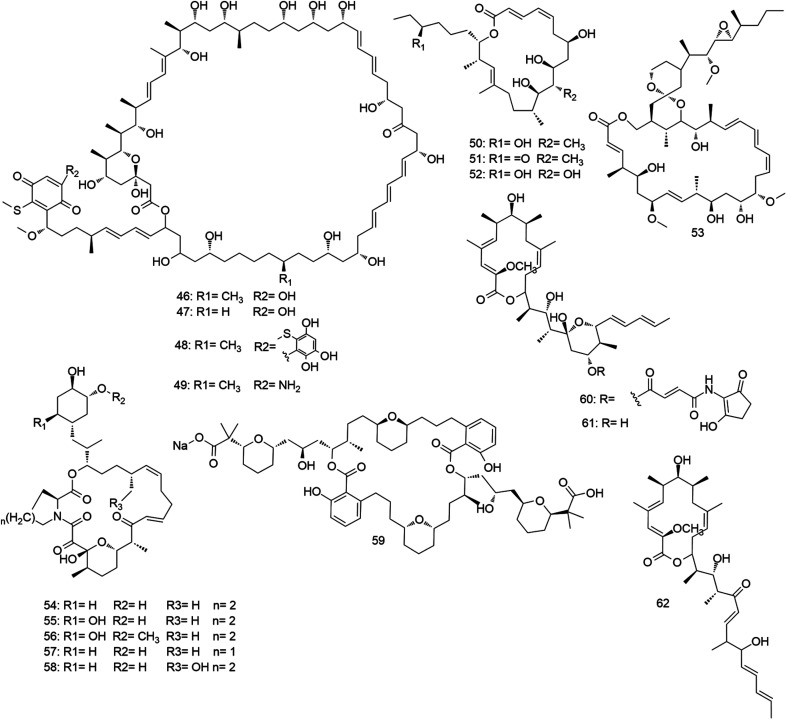
Macrolides derived from *Micromonospora* with anticancer activity (46–53), and other miscellaneous macrolides (compounds 54–62).

**Fig. 7:**
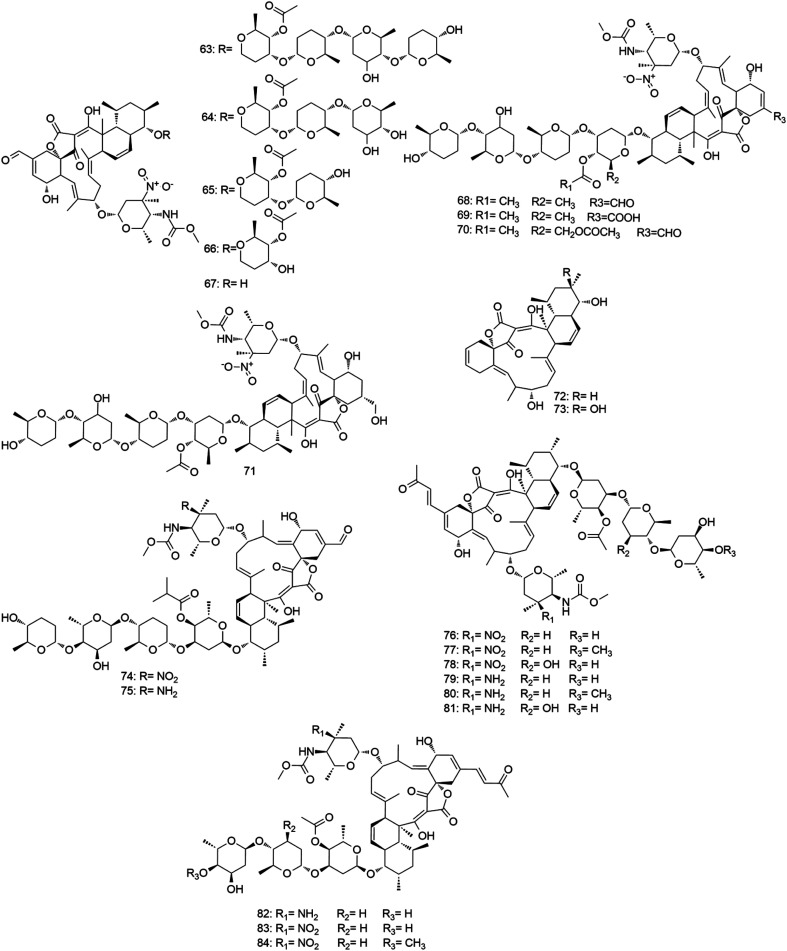
Unusual macrolides derived from *Micromonospora* (compounds 63–84).

Macrolides are regarded as one of the most efficient natural drug leads for treating immune and infectious diseases. They consist of large macrocyclic lactone ring (14, 15, or 16-membered) attached to one or more amino-deoxy sugars. Biosynthetically, macrolides are polyketide-derived natural products that utilize polyketide synthase (PKS) systems.^20^ Macrolide antibiotics display a bacteriostatic action against a wide range of pathogenic bacteria through binding to the 50S subunit of the bacterial rRNA complex.^21^ Macrolides are characteristic secondary metabolites for the genus *Micromonospora* (Fig. 5–7), where several examples from this class of specialized metabolites have been reported during the last fifty years.

#### 3.1.1. Antimicrobial macrolides

Megalomicin was the first macrolide antibiotic reported from *Micromonospora* in 1969. It was isolated from the soil-derived strain *M. megalomicea* as an antibiotic mixture of several antimicrobial substances which were identified as megalomicins A, B, C1, and C2 (1–4).^22^ In the same year, the structure of megalomicin A was elucidated.^23^ In 1979, its structure was revised after detailed X-ray crystallographic analysis.^24^ The biosynthesis of megalomicins resembles that of erythromycin C (5) which is glycosylated to the amino-deoxy sugar l-megosamine in the last step.^25^ Megalomicins exert bacteriostatic activity primarily against Gram-positive bacteria comparable to erythromycin (6) both *in vitro* and *in vivo*.^22^ Moreover, megalomicin C1 (3) revealed a good antiviral activity against swine fever virus and herpes simplex virus type 1 (HSV-1) by inhibiting the viral protein glycosylation.^26^ Later, megalomicin A (1) was found to exhibit potent to moderate antiparasitic activities (IC_50_ 0.2, 1, 2, 3, and 8 μg mL^−1^) when tested against *Trypanosoma cruzi*, *Plasmodium falciparum*, *Trypanosoma brucei*, *Leishmania donovani*, and *Leishmania major*, respectively. Megalomicin A (1) uniquely blocks vesicular transport between the medial- and trans-Golgi, resulting in the under-sialylation of the parasite proteins.^27^ Rosamicin (7), another example of macrolide antibiotics isolated from *M. rosaria*^12^ that is structurally more related to cirramycin A1 (8) than erythromycin (6). Rosamicin (7) demonstrates broad antibiotic activities, particularly against Gram-negative bacteria. Besides, it has superior stability under acidic conditions in comparison to megalomicins or erythromycins. The rosamicin aglycone rosaramcin (9) along with its diacetate (10), that exhibited the same antibiotic potency and spectrum as rosamicin (7), were prepared *via* the asymmetric total synthesis in 1986.^28^ One year later, during the screening for new antibiotics produced by actinomycetes collected at Izena Island, Okinawa, Japan, three rosamicin analogues designated as izenamicins A3, B2, and B3 (11–13) were isolated from a soil-derived *Micromonospora* strain. Only izenamicin B3 (12) showed potent antibacterial effects against a panel of Gram-positive and Gram-negative pathogenic bacteria.^29^ From a soil strain *M. chalea*, a group of seven 16-membered macrolide antibiotics structurally close to rosamicin (7) were reported as juvenimicins A1–A4 (14–17) and juvenimicin B1–B3 (18–20). They demonstrated antibacterial activity against Gram-positive bacteria, particularly juvenimicin A3 (16) which was the most potent one (MIC 0.1–0.3 μg mL^−1^).^30^ Ten years later, another group of macrolide aglycones named rustmicin (21) and neorustmicin A–D (22–25) with potent antifungal activity against wheat stem rust fungus was obtained from the fermentation broth of the same *Micromonospora* species.^31–33^ Later in 1998, it was found that rustamicin (18) and its congeners mediate their fungicidal activity *via* inhibition of the fungal inositol phosphoceramide synthase resulting in interrupted sphingolipid biosynthesis.^34^ In 1980, further eighteen compounds called mycinamicin I–XVIII (26–43) were added to the 16-membered macrolide antibiotics family from a soil derived-*M. griseorubida* strain. Interestingly, mycinamicins showed potent bacteriostatic activity against Gram-positive pathogenic bacteria (MIC 0.1–3.12 μg mL^−1^).^35,36^ After eight years, both mycinamicin IV (29) and VII (32) were synthesized.^37^ Later, a biosynthetic pathway for this class of macrolides was proposed depending on the several biosynthetic intermediates that were isolated, particularly mycinonic acid (44), in addition to many of bioconversion studies.^38^ In 2003, their biosynthetic gene cluster was completely determined in *M. griseorubida* suggesting that some genes in the cluster might have been horizontally transferred from *Streptomyces* spp.^39^ The large non-polyene macrolide perimycin (45) was first isolated from *Streptomyces primycini* and showed potent antibiotic activity towards Gram-positive bacteria.^40^ Later, it was obtained in a higher quantity from *M. galareinsis*.^41^ Perimycin (45) has a unique macrolide scaffold with attached guanidine and arabinose moieties that enables it to target the bacterial cell membrane and alter its permeability.^42^ In 2014, this interesting antibiotic was re-evaluated against prevalent multi-resistant Gram-positive bacteria responsible for common skin infections.^43^ Besides its promising antibiotic activities, perimycin (45) possesses significant antifungal effects against a wide range of pathogenic fungi.^44^ Its complex formation with the fungal cell membrane ergosterol is considered the primary mode of action responsible for its antifungal properties.^45^ All *Micromonospora*-derived antimicrobial macrolides are depicted in Fig. 5.

#### 3.1.2. Anticancer macrolides

Besides their significant antimicrobial activities, macrolides derived from *Micromonospora* have demonstrated considerable *in vitro* antiproliferative effects towards various human cancer cell lines. The first anticancer macrolide recovered from *Micromonospora* was in 1993, when the 60-membered polyene macrolides quinolidomicins A1, A2 and B1 (46–48) were isolated and characterized from a soil-derived *M. chalcea*.^46^ Both quinolidomicins A1 and B1 (46, 48) demonstrated significantly potent *in vitro* antiproliferative activity (IC_50_ 25–327 ng mL^−1^) toward an array of human cancer cell lines including multidrug-resistant cells.^46^ Quinolidomicins are considered the largest macrolide of terrestrial origin identified to date. Recently, their biosynthetic gene cluster was characterized *via* heterologous expression to be over 200 kb (154 domains in 34 modules). Detailed chemical characterization of the product led to a structural revision, in which the hydroxy group in the quinone moiety of quinolidomicin A1 (46) was replaced by an amino group (49).^47^ In 2011, while exploring the diversity of actinomycetes in the deep-Mediterranean seafloor, a new *Micromonospora* strain was found to produce two new 20-membered macrolides, levantilide A and B (50, 51).^48^ Two years later, levantilide C (52) was isolated from another *Micromonospora* strain that was isolated from shallow coastal waters near the island of Chiloe, Chile.^49^ All reported levantilides showed significant *in vitro* antiproliferative activity against different human cancer cell lines (2.6–8.3 μg mL^−1^).^48,49^ In 2018, utilizing bioinformatic approaches together with spectroscopic tools led to the isolation and full characterization of neaumycin B (53), a complex polycyclic macrolide with 19 stereo centers from a marine-derived *Micromonospora* strain. Structural characterization of such complex compounds requires selective degradation, crystallization, derivatization, X-ray diffraction analysis, total asymmetric synthesis, or other time-consuming methods to assign the complete stereo-structure. Alternatively, the genome of the producing microbe was sequenced to identify the gene cluster of neaumycin (*neu*). By integrating the known stereospecific biosynthetic enzymes with comprehensive NMR analysis, the full stereo-structure of neaumycin B (53) was characterized. This unique metabolite revealed potent and selective *in vitro* inhibitory activity against glioblastoma cell line indicating it as a promising drug candidate.^50^ All anticancer *Micromonospora*-derived macrolides are depicted in Fig. 6.

#### 3.1.3. Macrolides with other biological activities

In 1996, bio-guided chemical investigation of a soil-derived *Micromonospora* strain led to the isolation of five novel ascomycin-like macrolides, antascomicins A–E (54–58). They were found to bind strongly to the rapamycin-binding protein FKBP12 (IC_50_ 0.7 ng mL^−1^) and antagonize the immunosuppressive activity of rapamycin.^51^ These interesting effects of antascomicins family prompted a group of organic chemists in 2005 to initiate an antascomicins total synthesis campaign as potential ligands for FKBP12 protein that could promote the growth of damaged nerves in the peripheral nervous system without immunosuppressive side effects. They managed to totally synthesize antascomicin B (55) in 52 steps from commercially available starting materials.^52^ Later, another group reported the total asymmetric synthesis of the C_1_–C_21_ and C_22_–C_34_ fragments of antascomicin A (54).^53,54^ Searching for natural cholesterol-lowering drug leads, the ethyl acetate extract of a soil-derived *Micromonospora* strain displayed significant activity as an activator of the LDL-receptor gene promoter. LDL-receptor (LDL-R) mediates LDL cellular uptake and hence, reducing serum cholesterol level. Bio-guided fractionation of this extract led to the isolation of the selective LDL-R promoter activator, SCH 351448 (59).^55^ Later, several efforts for the enantioselective synthesis of this promising molecule were reported.^56–58^ In the course of the search for new inhibitors of starfish (*Asterina pectinifera*) embryonic development, three new bafilomycin-type 16-membered macrolides micromonospolides A–C (60–62) were isolated from the fermentation broth of a marine-derived *Micromonospora* strain. They were able to inhibit gastrulation of starfish embryos at MIC of 0.01, 0.011, and 1.6 μg mL^−1^, respectively.^59^ Miscellaneous macrolides (54–62) are depicted in Fig. 6.

#### 3.1.4. Unusual macrolides: spirotetronates

Spirotetronates are a unique family of complicated macrolide that features tetronic acid (spiro-linked to a cyclohexene ring) linked to a trans-decalin moiety. The structure is usually connected to two sugar moieties, of which one is a nitro sugar, d-tetronitrose, and the other one is a chain of deoxy sugars, l-digitoxoses and l-amicetoses. In terms of biological effects, this class of compounds exhibits wide-range of biological activities, including antitumor, antibacterial and antiviral activities. Tetrocarcins A–C (63–65) were the first representatives of spirotetronates in 1980 and obtained in a high yield from *M. chalcea*.^60^ Later, tetrocarcins E and F (66, 67) were reported from the same strain.^61^ All the reported tetrocarcins demonstrated significant antibacterial effect against Gram-positive bacteria (MIC 0.1–30 μg mL^−1^), and potent *in vivo* antitumor effects against different human breast cancer.^60,61^ In 2018, tetrocarcins AC6H, N, H and Q (68–71) were isolated from the marine-derived strain *M. carbonacea*.^62^ In 1991, a group of Japanese chemists has achieved the first total synthesis of tetrocarcins aglycone (72, 73) *via* coupling of spirotetronate with octahydronaphthalene aldehyde.^63^ Tetrocarcins mediate their antibacterial activity through the inhibition of bacterial DNA dependent RNA polymerase, and this activity is decreased with the decrease in the number of deoxy sugars attached to the aglycone.^64^ Moreover, they mediate their anticancer effect through induction of apoptosis in cancer cells by inactivation of phosphatidylinositide-3′-kinase-dependent pathway.^65^ In 2000, another example of spirotetronates, with an iso-butanoyl digitoxose unit instead of the acetyl digitoxose, arisostatins A and B (74, 75) that were isolated from a halophilic *Micromonospora* strain. Both metabolites demonstrated biological activity like their parent spirotetronates, tetrocarcins.^66^ Recently, a marine-derived strain *M. harpali* was found to produce a group of new spirotetronates analogs (76–84) that displayed strong to moderate antibacterial activities against Gram-positive bacteria (MIC 0.016–8 μg mL^−1^).^67^

### 3.2. N-containing metabolites

#### 3.2.1. Aminoglycoside antibiotics

**Fig. 8:**
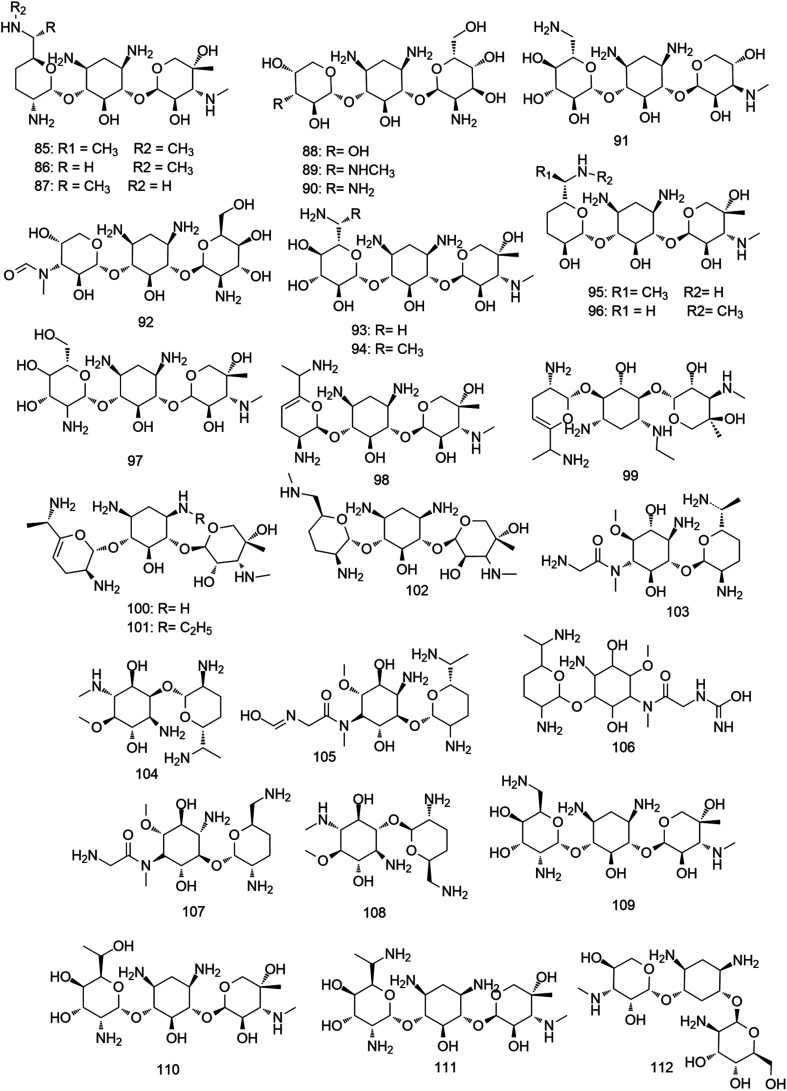
Aminoglycoside antibiotics derived from *Micromonospora* (compounds 85–112).

Aminoglycosides are one of the oldest classes of antimicrobial agents. They were mainly reported from either soil-derived *Streptomyces* or *Micromonospora* species (Fig. 8).^68^ Generally, the aminoglycoside molecule consists of two or more amino sugars connected to an aminocyclitol nucleus. They have broad-spectrum antibacterial activity against Gram-positive and Gram-negative bacteria, mycobacteria, and protozoa.^69^ Their mode of action is mediated by binding to the aminoacyl site (A-site) of the bacterial 16S ribosomal RNA (rRNA), where they interrupt the “proof-reading” process that ensures the accuracy of protein synthesis, in addition to the 50S ribosomal subunit, which inhibits the translocation and ribosome recycling.^70,71^ Investigation of the biosynthetic origins and pathways for this vital class of antibiotics has remarkably developed during the past decade. Biosynthetic gene clusters of different aminoglycoside derivatives including gentamicin have been identified to date. The excellent review of Kudo and Eguchi summarized the latest advances in the characterization of aminoglycosides biosynthetic genes.^72^

Gentamicin complex was the first reported aminoglycoside from *Micromonospora* in 1963.^73^ This 4,6-disubstituted aminocyclitol antibiotic complex is mainly composed of three major components; gentamicin C1, C1a and C2, and were isolated from two strains: *M. echinospora* NRRL 2953 and *M. echinospora* NRRL 2985 from a soil sample in New York, USA. The C1, C1a and C2 (85–87) components are composed of a central 2-deoxystreptamine (di-aminocyclitol) 4,6-disubstituted with the secondary sugars garosamine and purpurosamine. On the other hand, different trace components, such as gentamicins A, A1, A2, A3, A4, B, B1 (88–94) were found to be produced by several gentamicin producing *Micromonospora* strains. Gentamicins have shown potent antibacterial effect against a panel of Gram-positive and Gram-negative pathogenic bacteria both *in vitro* (MIC 0.1–10 μg mL^−1^) and *in vivo*. In addition, they exhibited excellent pharmacokinetic properties and moderate tolerability within their therapeutic dose. So, they were successfully introduced into the pharmaceutical market in 1971 and from this time, they were used for diverse medical applications. Gentamicins successful story has encouraged further aminoglycosides exploration from *Micromonospora*.^74^ In 1974, Sisomicin (95), an aminoglycoside antibiotic that differs from gentamicins by having unsaturated sugar ring, was isolated from *M. inyoensis* NRRL 3292. This antibiotic, in contrast to gentamicin, is produced substantially as a single component and showed interesting antibacterial effect against both Gram-positive and negative bacteria with MIC values ranged from 0.01–7.5 μg mL^−1^.^75^ It displayed better antibacterial effect compared to other structurally-related aminoglycoside molecules such as gentamicin, tobramycin, and amikacin, which could be related to the minor difference found in the ring I of sisomicin (95).^76^ Sisomicin (95) has also shown to be an attractive molecule to design and modify semisynthetic aminoglycoside analogues not only due to its potent antibacterial activity but also its resistance to numerous aminoglycoside-modifying enzymes, such as aminoglycoside phosphotransferases (APHs) and aminoglycosides nucleotidyltransferases (ANTs), by lacking the 3′- and 4′-OH groups in ring I.^77^ Mutamycin (96), a sisomicin (95) analogue, was found to be produced upon addition of 2-deoxystreptamine to the fermentation broth of *M. inyoensis*. It has shown antibacterial activity similar to sisomicin (95) (MIC 0.08–3 μg mL^−1^).^78^ Additionally, sagamicin (97) was isolated from the fermentation broth of *M. sagamiensis* MK 62, and demonstrated antibacterial activity against Gram-positive and negative bacteria similar to gentamicin C1 (85) with MIC values of 0.01–8.3 μg mL^−1^.^79^ Sagamicin (97) was considered one of the most effective agents against *Enterobacteriaceae* (MIC 2 μg mL^−1^) and displayed higher antipseudomonal activity than that of gentamicins.^80^ A year after, verdamicin (98) along with its 1-*N*-ethyl derivative, vertilmicin (99) were isolated from both *M. grisea* and *M. olivoasterospora*, respectively, with antibacterial activity against Gram-positive and Gram-negative bacteria (MIC 0.03–3.0 μg mL^−1^) similar to that of sisomicin (95) and gentamicin C1 (85).^81,82^ In 1977, fortimicins A–C (100–102) were produced as a mixture of several biologically active components from *M. olivoasterospora* as usually the case with aminoglycoside antibiotic fermentations.^83^ Fortimicin A (100) exhibited broad-spectrum potent antibacterial effects against Gram-positive and negative bacteria both *in vitro* (MIC 0.02–10 μg mL^−1^) and *in vivo*, while fortimicin B and C (101, 102) were less active.^84^ From the same *Micromonospora* strain, antibiotic SF-1854 was isolated as a byproduct and characterized as 1, *N*-formylfortimicin A (103). It showed 8–16 times weaker activity than fortimicin A (100). However, its activity against *Salmonella*, *Pseudomonas* and *Streptococcus faecalis* was relatively close to fortimicin A (100) with MIC values ranged from 6.25–50 μg ml^−1^.^85^ Later, further fortimicins C, D and K (104–106) were isolated from the same strain. Fortimicins C and D (104, 105) showed more potent antibacterial activity against Gram-positive and negative bacteria than fortimicin A (100) with MIC values of 0.02–0.33 μg mL^−1^.^86^ In the same year, antibiotic G-418 (107) and antibiotic JI-20A (108) were isolated from *M. rhodorangea* NRRL 5326 and *M. purpurea* JI-20, respectively. They exhibited broad-spectrum antibacterial activity with MIC values of 16–64 μg mL^−1^. Moreover, they showed significant activity against protozoa, amoebae, tapeworm, and pinworm infections in mice.^87^*M. zionensis* was found to produce a broad-spectrum aminoglycoside antibiotic named G-52 (109) with potent activity against both Gram-positive and negative bacteria with MIC values ranged from 0.01–17.5 μg mL^−1^.^88,89^ Furthermore, antlermicin A–C (110–112), were isolated from the culture of *M. chalcea*. They showed strong antibacterial activity against *Bacillus subtilis* with MIC values of 0.015, 0.05, 0.039 μg mL^−1^, respectively. In contrast to the other aminoglycosides, they did not exhibit any activity against Gram-negative bacteria. However, they inhibited the growth of sarcoma cells *in vitro* with IC_50_ values of 1, 1.56, 12.5 μg mL^−1^, respectively.^90,91^ All *Micromonospora*-derived aminoglycosides are depicted in Fig. 8.

#### 3.2.2. Anticancer alkaloids

**Fig. 9:**
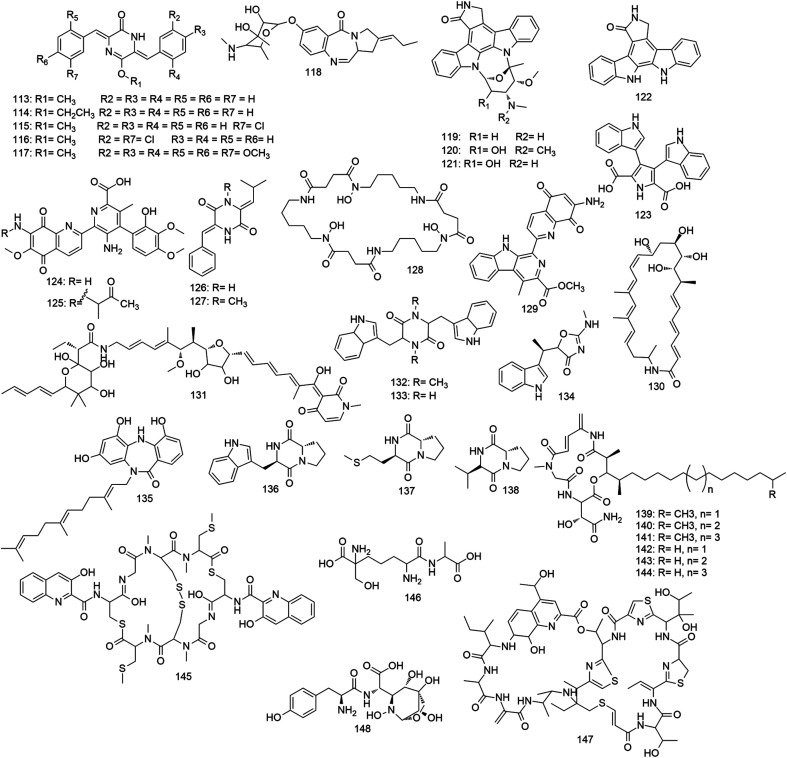
Other N-containing metabolites derived from *Micromonospora*. Compounds 113–131 are alkaloids with anticancer activity, compounds 132–138 are alkaloids with antimicrobial activity, compounds 139–145 are peptides with anticancer activity, compounds 146–148 are peptides with antimicrobial activity.

Alkaloids are class of naturally occurring nitrogenous organic compounds. They are produced by a large variety of terrestrial and marine organisms as well as microorganisms including actinomycetes. Most alkaloids derived from *Micromonospora* species (Fig. 9) showed promising anticancer and antimicrobial activities. In 1988, Yang *et al.* reported the first alkaloid from the genus *Micromonospora*. During their chemical investigation of the soil-derived strain *M. neihuensis*, they have isolated a piperazine-type alkaloid, neihumicin (113) which demonstrated potent *in vitro* antiproliferative effect toward KB cell line (IC_50_ 0.94 μg mL^−1^).^92^ Later, they papered a group of semi-synthetic analogues (114–117) to study their structure–activity relationship which indicated that the presence of an alkyl ether group together with the substitution at the two benzene rings are essential for the cytotoxic effect of these molecules (IC_50_ 0.08–0.94 μg mL^−1^).^93^ A new member of the anthramycin antibiotics family, sibanomicin (118) was identified from the culture filtrate of a marine-derived *Micromonospora* strain. Sibanomicin (118) showed significant *in vivo* antitumor activity in mice bearing leukemia P388 cells.^94^ In 2000, bioprospecting of the marine sponge-associated actinomycetes led to the identification of staurosporine (119)-producing *Micromonospora* strain. Large scale fermentation of this new strain afforded further two indolocarbazole derivatives, 4′-*N*-methyl-5′-hydroxystaurosporine (120) and 5′-hydroxystaurosporine (121). The isolated alkaloids showed potent *in vitro* antiproliferative activity against a panel of human cancer cell lines (IC_50_ 0.001–0.04 μg mL^−1^) as a result of inhibition of the protein kinase C (IC_50_ 44 ng mL^−1^).^95^ Later, staurosporine (119) has shown to induce apoptosis in various cancer lines by activating caspase-3.^96^ Recently, staurosporine (119) along with its congeners are considered non-selective kinase inhibitors, and this lack of specificity has precluded their clinical use, however, they became important compounds to be used frequently for various biochemical experiments.^97^ Early 1990s, a number of synthetic approaches for staurosporine (119) and its aglycone (122) were described in different reports.^98–100^ The biosynthesis of staurosporine (119) and its analogues was proposed to start with the amino acid l-tryptophan which in turn converted to an imine by the oxidase enzyme StaO. Subsequently, two imine groups are dimerized to afford chromopyrrolic acid (123). Finally, an aryl–aryl coupling followed by decarboxylation reaction proposed to be catalyzed by a cytochrome P450 enzyme leads to the formation of staurosporine aglycone (122).^101^ In 2002, staurosporine (119) biosynthetic gene cluster was cloned and successfully expressed in *Streptomyces lividans*.^102^ The aminoquinone alkaloid streptonigrin (124) was originally isolated from *Streptomyces flocculus* in 1963, and again in 2002 when Wang and his co-workers reported a soil-derived *Micromonospora* strain that produced streptonigrin (124) in large quantities along with its oxopropyl analogue (125). Afterward, several synthetic chemists have developed different methods for the total synthesis of this unique alkaloid (124).^103–105^ Moreover, the piperidine alkaloids, albonoursin (126) and its *N*-methyl derivative (127) together with the siderophore norcardamine (128) were also recovered from the same *Micromonospora* strain fermentation broth. Both streptonigrin (124) and its analogue (125) were able to induce apoptosis in the human neuroblastoma SH-SY5Y cells through p53-dependent mechanism.^106^ Recently, its biosynthetic gene cluster (48 genes) was fully characterized proposing a novel enzymatic reactions pathway where lavendamycin methyl ester (129) is formed and subsequently an aromatic dioxygenase StnB1/B2 system catalyzes a regiospecific cleavage of the N–C8′ bond of the lavendamycin methyl ester (129) indole ring.^107^ In 2018, the bioactivity-guided chemical profiling of the *Micromonospora* strain recovered from the East China Sea sediment led to the isolation of a new 26-membered polyene macrolactam compound FW05328-1 (130), in addition to a known pyridine-derived polyene, aurodox (131). Both metabolites exhibited considerable *in vitro* antiproliferative activities against a number of human tumor cell lines with IC_50_ range 0.002–30.7 μg mL^−1^.^108^

#### 3.2.3. Antimicrobial alkaloids

Similarly, several antimicrobial alkaloids with diverse chemical structures were reported from different species of the genus *Micromonospora*. In 2004, three diketopiperazine alkaloids (Sch 725418, fellutanine, and indolmycin) (132–134) with moderate antibacterial and antifungal properties were isolated from a new *Micromonospora* strain.^109^ In the same year, a novel dibenzodiazepine alkaloid, diazepinomicin (135), was isolated from a marine-derived *Micromonospora* species. Diazepinomicin (135) represents a unique class comprising a rare dibenzodiazepine core conjugated to a farnesyl side chain. It exhibited modest antimicrobial activity against many pathogenic Gram-positive bacteria with MICs of about 32 μg mL^−1^.^110^ Besides, diazepinomicin (135) demonstrated broad *in vitro* antiproliferative activity against a panel of cancer cell lines and *in vivo* efficacy in xenograft tumor models.^111^ Preclinical data suggested that diazepinomicin (135) is a promising anticancer agent with a dual-mode of action; selective binding to the peripheral benzodiazepine receptors and inhibition of the Ras–MAPK pathway.^112^ Furthermore, characterization of its biosynthetic gene cluster highlighted the enzymatic complexity needed to produce such structural type which is unprecedented among microbial metabolites.^113^ Recently, some diketopiperazines (136–138) with weak antibacterial activity (128 μg mL^−1^) were recovered from a sponge-associated *Micromonospora* strain. All *Micromonospora*-derived alkaloids are depicted in Fig. 9.

#### 3.2.4. Anticancer peptides

**Fig. 10:**
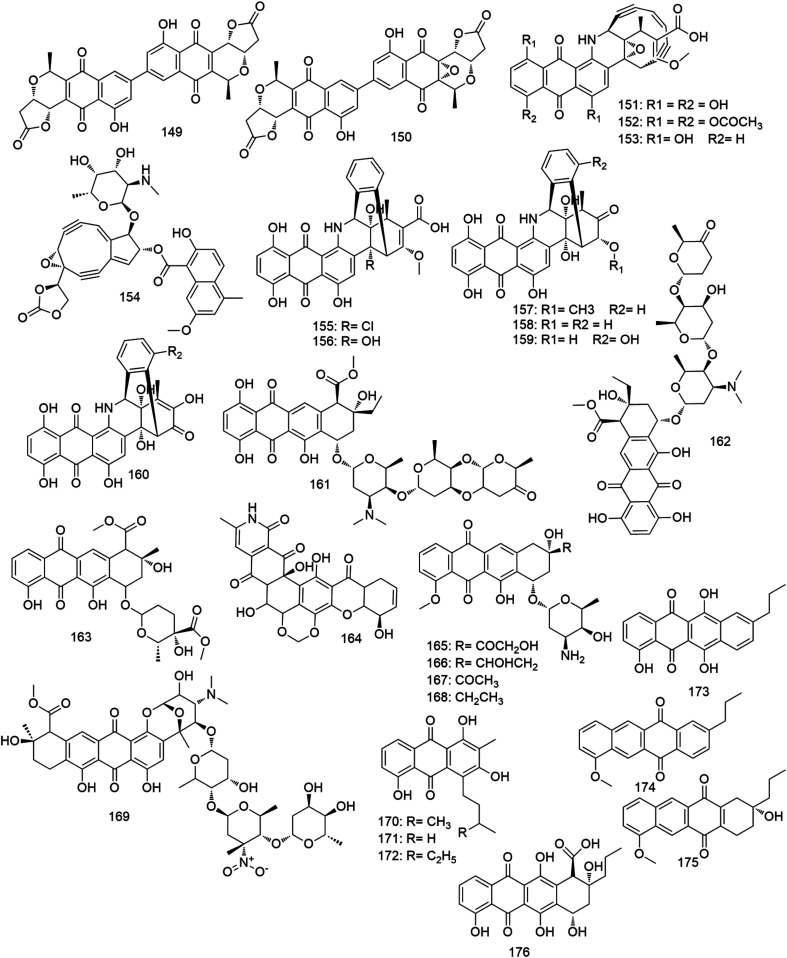
Quinones derived from *Micromonospora*. Compounds 149–164 have antimicrobial activities, and compounds 165–176 have anticancer activity.

Peptides are short chains of amino acid monomers linked through peptide bonds, which formed once the carboxyl group of an amino acid reacts with the amino group of another one. Peptides are classified according to their biosynthetic origin to ribosomal and non-ribosomal peptides. Ribosomal peptides in higher organisms are the products of a cellular ribosome, and functioning as hormones and signaling molecules. Some microorganisms produce ribosomal peptides as antibiotics.^114^ On the other hand, non-ribosomal peptides are assembled by enzymes complexes. They are common in unicellular organisms, fungi and bacteria and biosynthesized through non-ribosomal peptide synthetases complex. These complexes contain many different modules to perform diverse chemical manipulations on developing the product. These peptides are often cyclic and can have highly complex structures.^115^ In the past twenty years, there were a number of reports on bioactive peptides produced by different strains of *Micromonospora* (Fig. 10). The very first of this group was discovered in 1995 when the lipopeptides rakicidins A and B (139, 140) were discovered from a soil-derived *Micromonospora* strain. Both lipo-tripeptides are cyclized through a β-hydroxy fatty acid and exhibited potent *in vitro* antiproliferative activity against M109 cell line (IC_50_ 55, 230 ng mL^−1^). Rakicidin B (140) differs from A (139) by one methylene group in the lipid side chain, indicating that the length of the lipid side chain may significantly affect the cytotoxic activity of this class of compounds.^116^ Later, a screening study on microbial natural products revealed that rakicidin A (139) triggers novel hypoxia-selective cell death in solid tumors through caspase-dependent and independent pathways.^117,118^ The absolute configuration of rakicidin A (139) was assigned through its total synthesis in 2014.^119^ Then, a structure–activity relationship study indicated that both the conjugated diene moiety together with the long alkyl chain are essential features for the unique cytotoxic activity of rakicidin A (139).^120^ Recently in 2018, further rakicidin analogues (E and G–I) (141–144) were recovered from the marine *M. chalcea* FIM 02-523.^121^ In 1997, a novel bioactive depsipeptide, thiocoraline (145), was characterized from the culture broth of a marine *Micromonospora* strain. This unique compound showed exceptional cytotoxicity against a panel of cancer cell lines (IC_50_ 0.002 μg mL^−1^) and strong antibacterial activity against Gram-positive bacteria (MIC 0.03 μg mL^−1^).^122^ Recently, thiocoraline (145) biosynthetic gene cluster was successfully characterized and expressed in two *Streptomyces* strains.^123^ After its challenging total synthesis, thiocoraline (145) was found to induce cell death by binding to DNA which led to cell necrosis.^124^ Peptides derived from *Micromonospora* are depicted in Fig. 9.

#### 3.2.5. Antibacterial peptides

Many antibacterial peptides were reported from different *Micromonospora* species (Fig. 10) in the past thirty years. In 1980, during an antibiotics screening program, a novel dipeptide, *N*-(2,6-diamino-6-hydroxymethylpimelyl)-l-alanine (146) was obtained from the culture broth of the marine-derived *M. chalcea*. This metabolite was able to inhibit the *in vitro* growth of *E. coli* by interference with the bacterial cell wall biosynthesis.^125^ Ten years later, an unusual sulfur-rich antibiotic, Sch 40832 (147) was isolated from the soil inhabitant strain *M. carbonacea*.^126^ Later, another unique dipeptide, SB-219383 (148) with selective and potent inhibitory activity towards the bacterial tyrosyl tRNA synthetase was isolated from a *Micromonospora* strain recovered from marine-sediment.^127^

### 3.3. Quinones

**Fig. 11:**
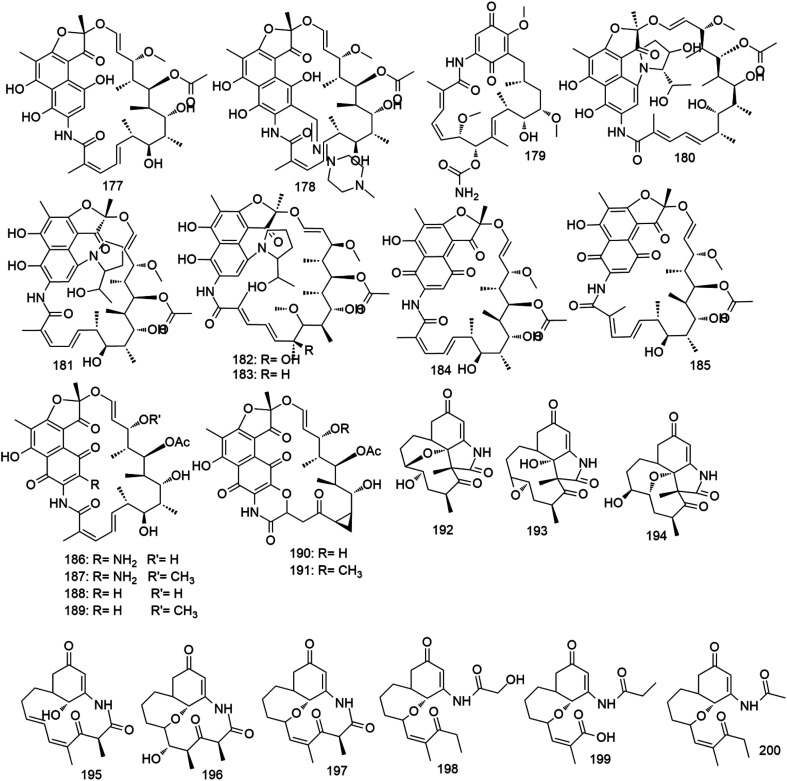
Ansamycins antibiotics derived from *Micromonospora* (compounds 177–200).

The quinones are a class of aromatic organic molecules derived from different aromatic nuclei such as benzene or naphthalene by the conversion of an even number of double bonds into ketone groups leading to a fully conjugated cyclic dione structure. *Micromonospora* species have proven to be a crucial source of medicinally important quinones (Fig. 11) in the last thirty years, in particular, the 9,10-anthraquinone class. Most of the *Micromonospora*-derived anthraquinones have found to be effective treatments for different types of malignant tumors and bacterial infections.^128^ Biosynthetically, these quinones originate from the polyketide pathway,^128^ and act on several biological targets including, DNA, topoisomerase II enzyme, and bacterial cell wall. Also, they can undergo intracellular redox reactions to generate excessive reactive oxygen species (ROS) that induces direct cell death. A number of *Micromonospora*-derived anthraquinones have reached the drug market, however, their adverse effects, particularly, cardiotoxicity, limited their use.^129^

#### 3.3.1. Antimicrobial quinones

In 1986, crisamicin A (149), derived from the halotolerant *M. purpureochromogenes*, was the first anthraquinone antibiotic reported. This isochromanequinone new member was active against a panel of Gram-positive pathogenic bacteria with MIC values ranged from 0.2–1 μg mL^−1^.^130^ Two years later, crisamicin C (150), the epoxide analogue of crisamicin A (149) was discovered from the same species and exhibited stronger antibacterial activity against Gram-positive bacteria (MIC 0.125–0.52 μg mL^−1^).^131^ In the same year, the biosynthesis of this class of compounds *via* the polyketide pathway was elucidated.^128^ Later in 2008, synthetic efforts resulted in the total asymmetric synthesis of crisamicin A (149).^132,133^ Dynemicin A (151), which was first isolated from the soil-derived *M. chersina*, featuring a unique hybrid of anthraquinone and an l,5-diyn-3-ene system, was found to have an extraordinary growth inhibitory effect against both Gram-positive (MIC 13–63 pg mL^−1^) and Gram-negative (MIC 63 μg mL^−1^) bacteria. Besides, its triacetate derivative (152) showed excellent *in vivo* therapeutic effect with considerable tolerability.^134^ Shortly after dynemicin A (151) discovery, another deoxy analogue (153) was isolated from *M. globosa* with similar antibacterial spectrum.^135^ Besides their outstanding antibiotic properties, dynemicins also demonstrated potent *in vitro* and *in vivo* anticancer effects against several human cancer cell lines (IC_50_ 0.1–1 μg mL^−1^).^134^ Dynemicins' primary mode of action is to intercalate into the minor groove of the DNA double helix by their enediyne moiety. Subsequently, chemically-induced conformational changes resulting in an irreversible double-stranded cleavage and immediate cell death. This mechanism is extremely cytotoxic due to the lack of any appropriate mechanism to repair a double-stranded cleavage of DNA in both prokaryotic and eukaryotic cells,^136^ and hence this prevents their further development as a drug. However, another *Streptomyces*-derived antibiotic known as neocarzinostatin (154) have the same enediyne functionality of dynemicins and act by the same mechanism, was approved for clinical use.^137^ The biosynthesis of dynemicin A (151) was proposed by connecting two heptaketide chains derived from seven head-to-tail joined acetate units.^138^ Later, a number of dynemicins (L–Q) (155–160) were further isolated from the dynemicin A producing organism *M. chersina.* They revealed much weaker antibacterial activity (MIC 0.08–1.25 μg mL^−1^) than their parent compound dynemicin A (151) due to the loss of the essential enediyne moiety.^139,140^ Spartanamicins A and B (161, 162), two antifungal antibiotics, were reported from a *Micromonospora* strain isolated from the medicinal plant *Asparagus officinalis*. Both anthracycline antibiotics have demonstrated potent antifungal activity (MIC 0.2–1 μg mL^−1^) against a wide range of pathogenic fungi.^141^ Besides, spartamicin B (162) has shown a promising *in vivo* activity against several multi-resistant fungal strains with no toxicity at the therapeutic dose (10 μg).^142^ In 2004, a marine-derived *Micromonospora* strain was found to produce a novel antimicrobial anthracycline, micromonomycin (163). This metabolite exhibited antibacterial activity against both Gram-positive and Gram-negative bacteria with MIC values range 0.5–4 μg mL^−1^. Additionally, it showed moderate antifungal properties with MIC values of 30–35 μg ml^−1^.^143^ Recently, a potent antiplasmodial polycyclic anthraquinone named MDN-0185 (164) was isolated from a soil-derived *Micromonospora* species. It was able to inhibit the growth of the multidrug-resistant *Plasmodium falciparum* with IC_50_ of 9 ng mL^−1^.^144^

#### 3.3.2. Anticancer quinones

In 1980, a number of daunorubicin derivatives (165–168) with potent *in vivo* antileukemic activity were recovered from an extremophilic *Micromonospora* strain.^145^ Later, a unique antitumor nitroanthracycline analog, cororubicin (169) derived from a marine *Micromonospora* strain, was able to inhibit the growth of many cancer cell lines (IC_50_ 0.8–4 μg mL^−1^) by generating intracellular active superoxide radicals.^146^ From the endophytic strain *M. lupini*, two new anthraquinones lupinacidins A and B (170, 171) were isolated. Both metabolites were found to effectively inhibit the invasion of murine colon 26-L5 carcinoma cells.^147^ Four years later, lupinacidin C (172) was isolated from the same *Micromonospora* strain and prepared by total synthesis. Additionally, it demonstrated the most potent inhibitory effects among the congeners on the invasion of colon cancer cells, indicating that the length of the alkyl side chain is essential for the anti-invasive activity.^148^ Recently, a tunicate associated *Micromonospora* strain, was found to produce some novel anthracyclinones (173–176) with moderate *in vitro* antiproliferative effects on the colon adenocarcinoma cells (IC_50_ 6–13 μg mL^−1^).^149^ All quinone-derived compounds that have been reported from *Micromonospora* are depicted in Fig. 10.

### 3.4. Ansamycins

**Fig. 12:**
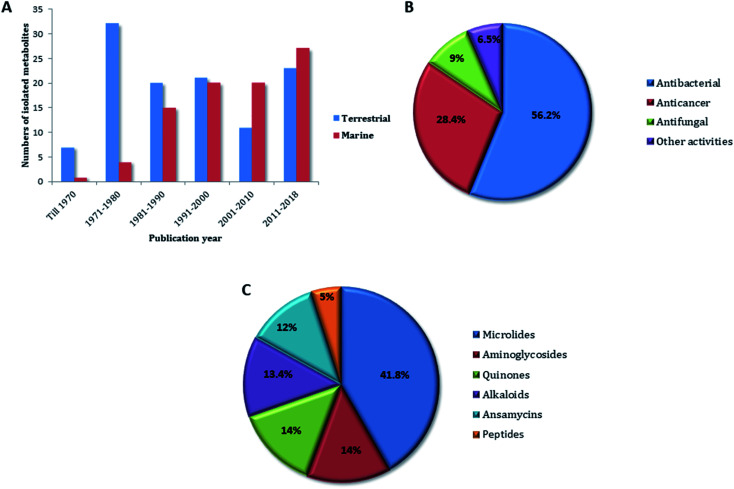
(A) Natural products isolated from genus *Micromonospora* according to the year of publication, (B) bioactivities of natural products derived from *Micromonospora* genus, (C) secondary metabolites classes derived from *Micromonospora* genus.

Ansamycins are a group of antibiotics with basket-like molecules that comprise an aromatic moiety (naphthalene or naphthoquinone ring) bridged at non-adjacent locations by an aliphatic chain. Their carbon skeleton is derived from the polyketide pathway *via* a polyketide synthase using an unusual starter unit.^150^ Rifamycin (177) was the first example in this class of antibiotics which was first isolated from *Streptomyces mediterranei* by Sensi and co-workers in 1959.^151^ Ansamycins show a broad spectrum of antibiotic activity against Gram-positive, particularly *Mycobacteria*, but to a lesser extent, Gram-negative bacteria. The antibacterial activity of ansamycins results from their ability to selectively inhibit the bacterial DNA-dependent RNA polymerase. There are several ansamycin related antibiotics reached the market *e.g.* rifampicin (178) and geldanamycin (179). During the last thirty years, several ansamycin antibiotics were reported from different *Micromonospora* species (Fig. 12), particularly the marine ones.

#### 3.4.1. Antibacterial ansamycins

Halomicins A–D (180–183) were the first example of *Micromonospora*-derived ansamycins. They were isolated from the marine strain *M. halophytica* and demonstrated potent antibacterial action against a wide range of Gram-positive bacteria (MIC 0.1–1 μg mL^−1^).^152^ In 2009, rifamycin S along with its geometric isomer (184, 185) were produced from *M. rifamycinica*, a marine sediment-derived new strain. They exhibited potent *in vitro* antibacterial activity against a panel of pathogenic Gram-positive bacteria including methicillin-resistant *Staphylococcus aureus* (MRSA) (MIC 0.03–0.25 μg mL^−1^).^153^ In 2017, a group of novel amino ansamycins, named sporalactams A–F (186–191) were isolated from another marine sediment-derived *Micromonospora* strain, and demonstrated very potent *in vitro* antibacterial effects against both Gram-positive and Gram-negative pathogenic bacteria including *Mycobacterium tuberculosis* (MIC 0.0008–0.06 μg mL^−1^).^154^ One year later, the activation of the positive regulator gene *mas13* induced the expression of the cryptic gene cluster *mas*, which was responsible for the biosynthesis of nine unusual pentaketide ansamycins, namely, microansamycins A–I (192–200) in a marine inhabitant *Micromonospora* species.^155^

## 4. Conclusions

Natural products derived from the genus *Micromonospora* possess a unique chemical diversity with huge therapeutic values making it an untapped resource of drugs and drug leads. Since its discovery almost 100 years ago, this genus has become a vigorous model for the discovery of naturally-derived drug leads. The collection of thousands of *Micromonospora* strains (Fig. 1) globally together with almost fifty years of research effort in both academia and industry has generated a resource that enabled to compare the biosynthesis of natural products among closely related environmental strains. This comparison has also revealed complications associated with microbial gene development and will continue to provide insights into the mechanisms of generating new structural diversity. Extensive biochemical investigation of *Micromonospora* has confirmed the concept that new microbial taxa that inhabit un- or under-explored environments such as marine habitats represent a promising source for natural product discovery. In general, the main source for the recovery and isolation of *Micromonospora* strains is soil (Fig. 2). Also, they were found as endophytic microorganisms associated with plant tissues. In marine ecosystems, they were isolated mainly from sediments, and marine organisms such as ascidians, sponges and soft corals. Additionally, there were few examples of talented *Micromonospora* species that inhabiting extreme environments (Fig. 2). Until 1980, soil-derived *Micromonospora* species were considered the main source of their specialized metabolites. Afterward, the interest in marine-derived species was increasing, and became a crucial source of chemical diversity especially in the last eight years (Fig. 12A). Most of the compounds identified from only 34% of the species reported in this genus (30 from 88 reported species). Additionally, its genomic analysis revealed a lot of unknown BGCs indicating their unprecedented potential for novel chemical leads discovery. Most of the *Micromonospora*-derived metabolites have shown excellent antimicrobial and anticancer effects (Fig. 12B), in particular, those belong to the macrolides, aminoglycosides, and ansamycins class of compounds (Fig. 12C). There are likely two major approaches to drug discovery from *Micromonospora* and other actinobacteria. The first and most common is cultivation dependent, in which isolated species are fermented, and subsequently, the produced secondary metabolites are recovered. Special requirements are needed not only for growth, but also for secondary metabolites the production makes this approach involves extensive effort. Besides, this cultivation-dependent technique is unable to unlock the full biosynthetic potential of such microorganism. The second approach is the genome-dependent bioprospecting in addition to chemoinformatic tools that can detect the silent biosynthetic gene clusters in the *Micromonospora*. Subsequent activation or heterologous expression of such gene clusters would potentially lead to the creation of robust pipelines for drug discovery from this genus. In conclusion, the continuous need for new bioactive molecules is of substantial importance. *Micromonospora* species provide a worthy platform for the discovery of novel biologically active compounds. More than 100 molecules have been reported from terrestrial strains including plant-associated ones. However, the less explored marine species along with those derived from extreme habitats afforded only 87 compounds, indicating a great potential for the discovery of novel therapeutic lead scaffolds upon the continuous investigation of *Micromonospora* from underexplored habitats in the future.

## Conflicts of interest

The authors declare no conflict of interest.

## Supplementary Material

RA-010-D0RA04025H-s001
