# Supplementary material for: The genus Micromonospora as a model microorganism for bioactive natural product discovery
Source: RSC Adv. 2020 Jun 8;10(35):20939–59. doi: 10.1039/d0ra04025h (PMC9054317; doi:10.1039/d0ra04025h)
Supplement: RA-010-D0RA04025H-s001 [file RA-010-D0RA04025H-s001.pdf]

Electronic Supplementary Material (ESI) for RSC Advances.
This journal is © The Royal Society of Chemistry 2020

The genus *Micromonospora* as a model microorganism for bioactive natural products discovery

Mohamed S. Hifnawy[1], Mohamed M. Fouda[2], Ahmed M. Sayed[2], Rabab Mohammed[3], Hossam M. Hassan[3], Sameh F. AbouZid[3], Mostafa E. Rateb[3,4], Alexander Keller[5], Martina Adamek[6,7], Nadine Ziemert[6,7,]*, and Usama Ramadan Abdelmohsen[8,9]*.

[1]*Department of Pharmacognosy, Faculty of Pharmacy, Cairo University, Cairo, Egypt 11787*

[2]*Department of Pharmacognosy, Faculty of Pharmacy, Nahda University, Beni-Suef, Egypt 62513*

[3]*Department of Pharmacognosy, Faculty of Pharmacy, Beni-Suef University, Beni-Suef, Egypt 62514*

[4]*School of Computing, Engineering and Physical Sciences, University of the West of Scotland, Paisley PA1 2BE, UK*

[5]*Center for Computational and Theoretical Biology, Biocenter, University of Würzburg, Hubland Nord, 97074 Würzburg, Germany*

[6]*Interfaculty Institute of Microbiology and Infection Medicine Tübingen, University of Tübingen, Tübingen, Germany*

[7]*German Centre for Infection Research (DZIF), Partner Site Tübingen, Tübingen, Germany*

[8]*Department of Pharmacognosy, Faculty of Pharmacy, Minia University, 61519 Minia, Egypt*

[9]*Department of Pharmacognosy, Faculty of Pharmacy, Deraya University, Universities Zone, P.O. Box 61111 New Minia City, 61519 Minia*

*Correspondence: usama.ramadan@mu.edu.eg (URA);
nadine.ziemert@uni-tuebingen.de (NZ)

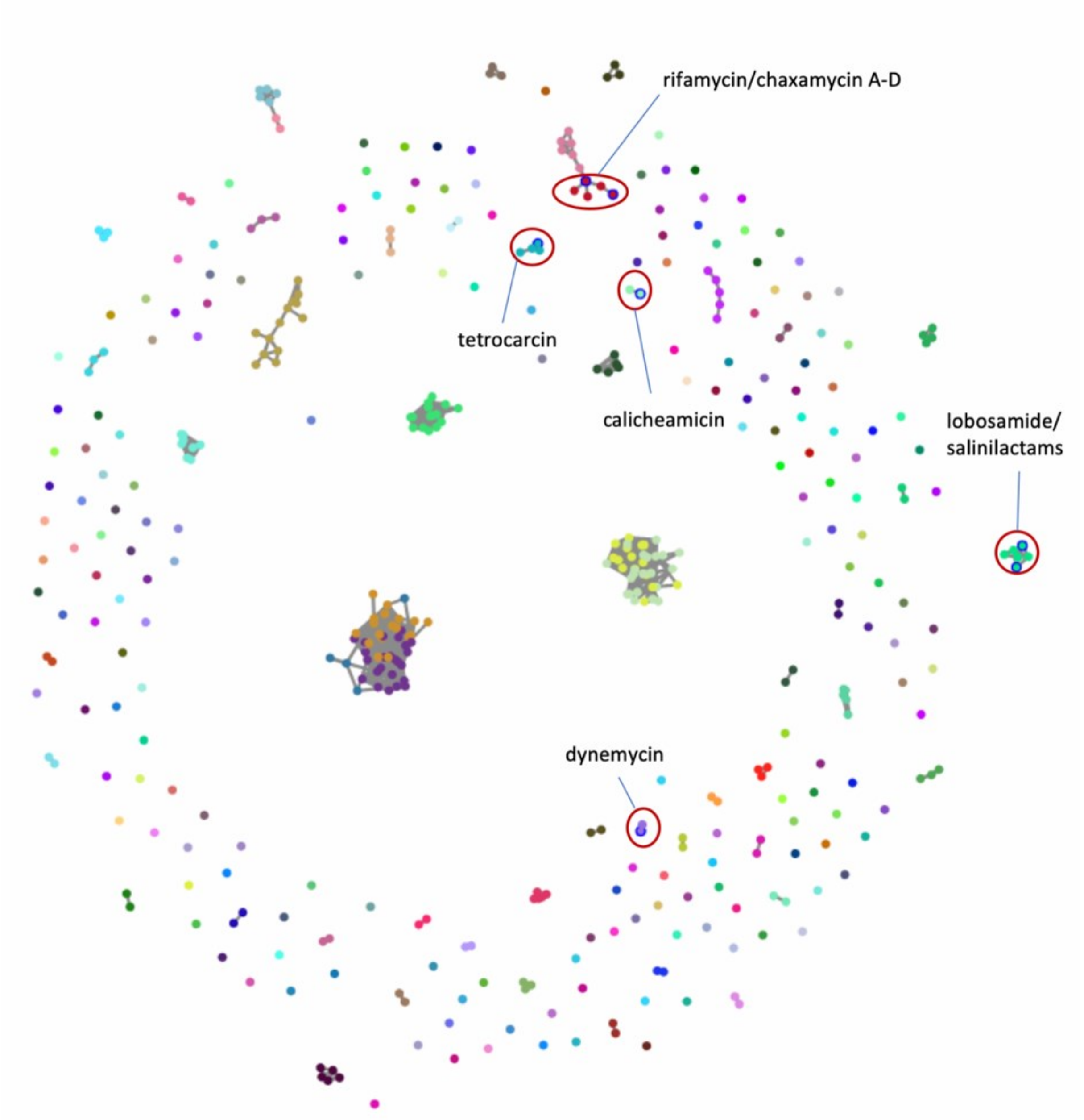

Figure S1: ***Micromonospora*** **- Biosynthetic Gene Cluster Similarity Networks of "Others" BGCs.**
Gene cluster similarity networks of PKS- BGCs generated with BiG-SCAPE from 87 *Micromonospora* genomes. Gene clusters were identified and classified using antiSMASH. Each node represents one sequenced gene cluster. Connected clusters likely encode for similar compounds. To identify already known and characterized BGCs, the dataset from the MIBiG database was added to the network analysis. MIBiG compounds are circled in red.

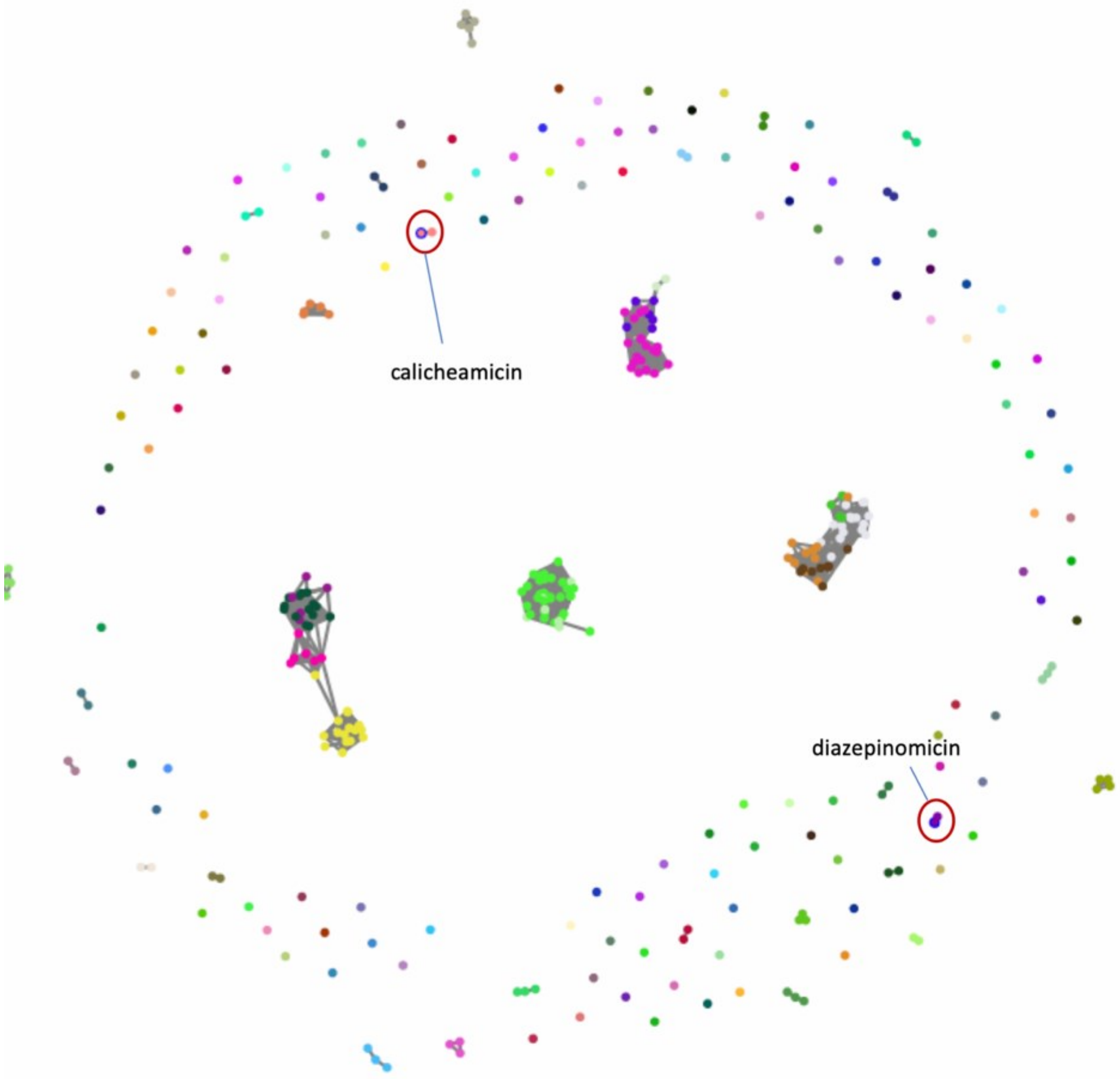

Figure S2: *Micromonospora* - Biosynthetic Gene Cluster Similarity Networks of "PKS I" BGCs

Gene cluster similarity networks of PKS- BGCs generated with BiG-SCAPE from 87 *Micromonospora* genomes. Gene clusters were identified and classified using antiSMASH. Each node represents one sequenced gene cluster. Connected clusters likely encode for similar compounds. To identify already known and characterized BGCs, the dataset from the MIBiG database was added to the network analysis. MIBiG compounds are circled in red.

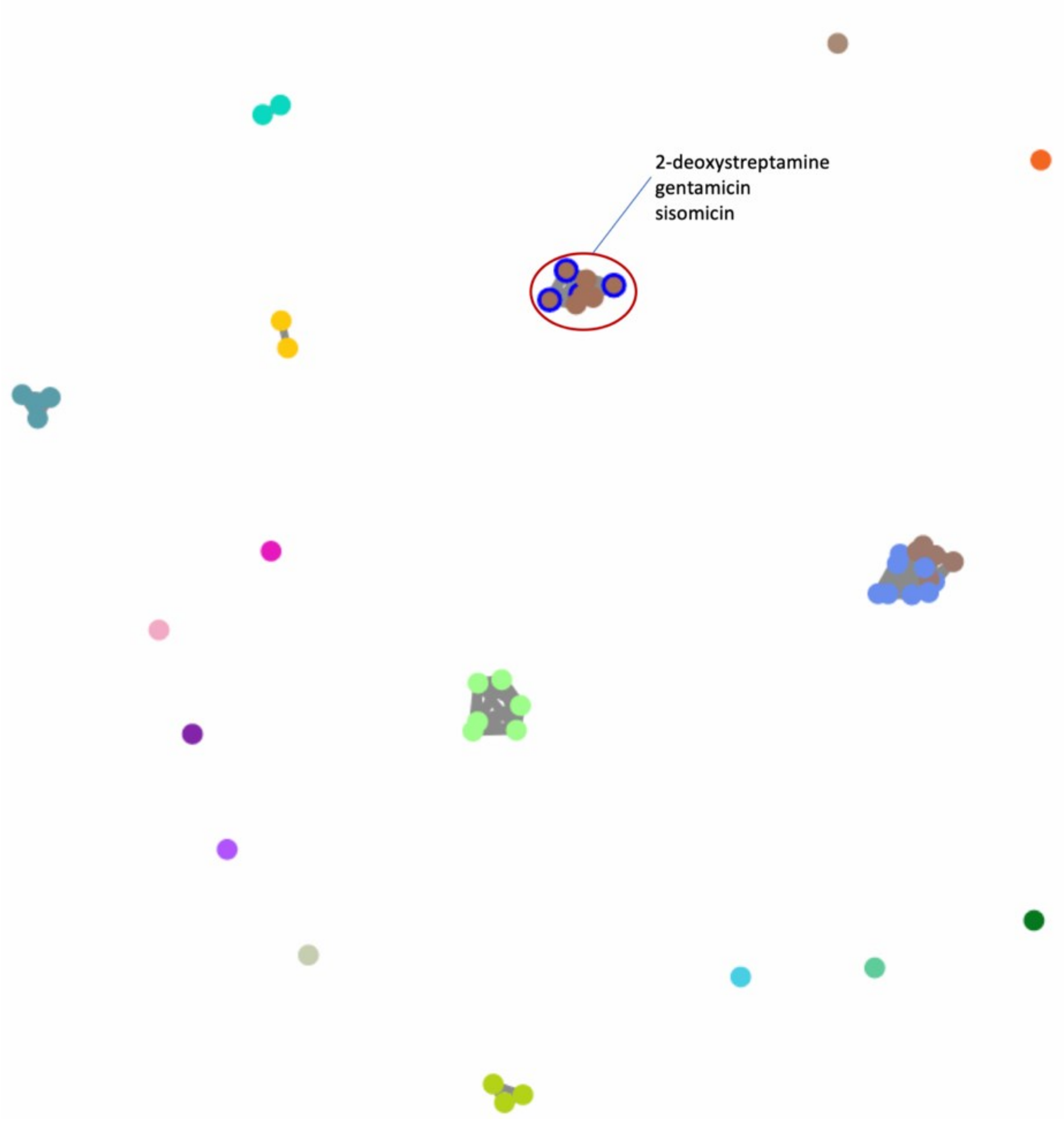

Figure S3: *Micromonospora* - Biosynthetic Gene Cluster Similarity Networks of "Saccharides" BGCs

Gene cluster similarity networks of PKS- BGCs generated with BiG-SCAPE from 87 *Micromonospora* genomes. Gene clusters were identified and classified using antiSMASH. Each node represents one sequenced gene cluster. Connected clusters likely encode for similar compounds. To identify already known and characterized BGCs, the dataset from the MIBiG database was added to the network analysis. MIBiG compounds are circled in red.

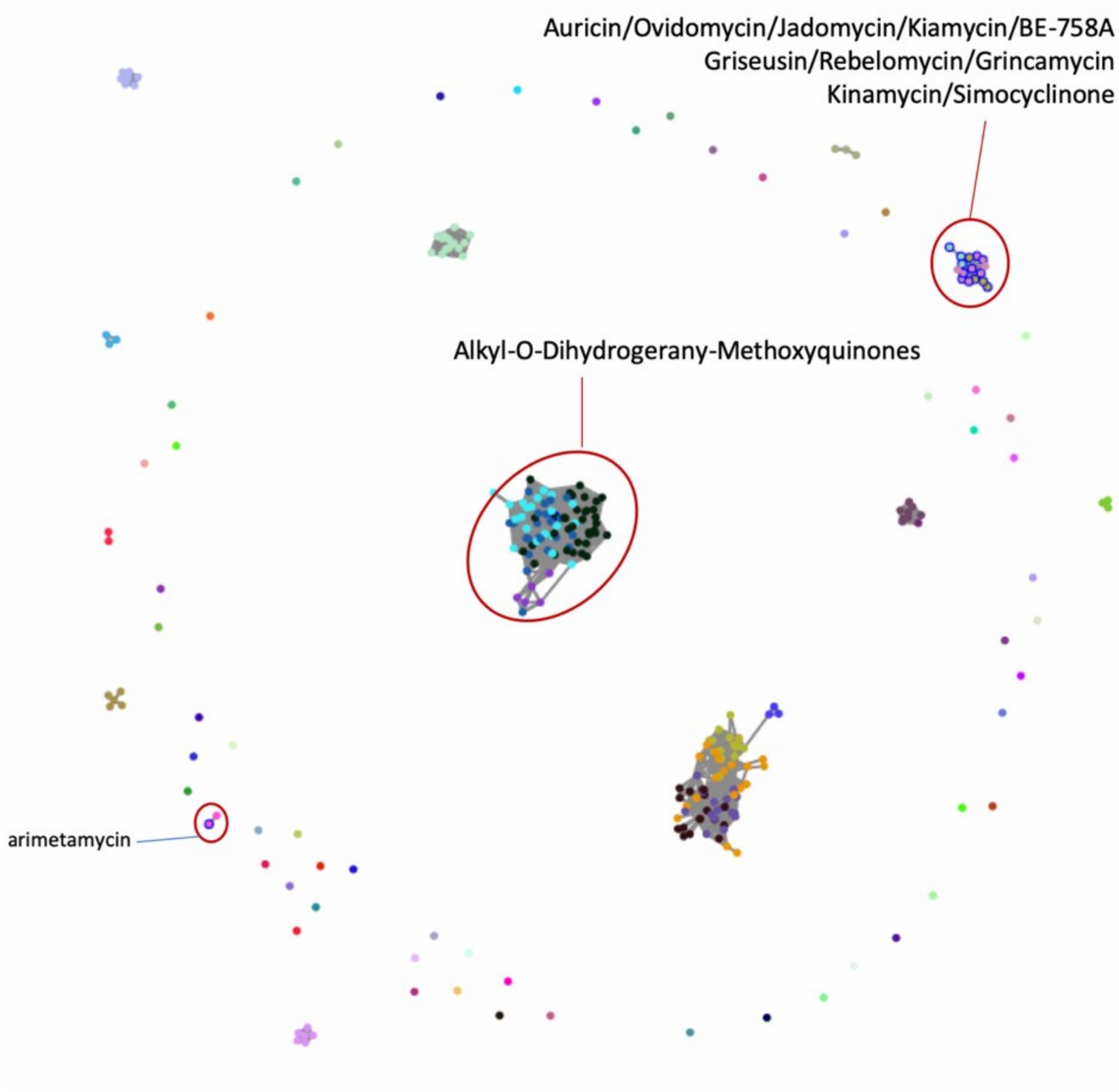

Figure S4: *Micromonospora* - Biosynthetic Gene Cluster Similarity Networks of "other polyketides" BGCs

Gene cluster similarity networks of PKS- BGCs generated with BiG-SCAPE from 87 *Micromonospora* genomes. Gene clusters were identified and classified using antiSMASH. Each node represents one sequenced gene cluster. Connected clusters likely encode for similar compounds. To identify already known and characterized BGCs, the dataset from the MIBiG database was added to the network analysis. MIBiG compounds are circled in red.

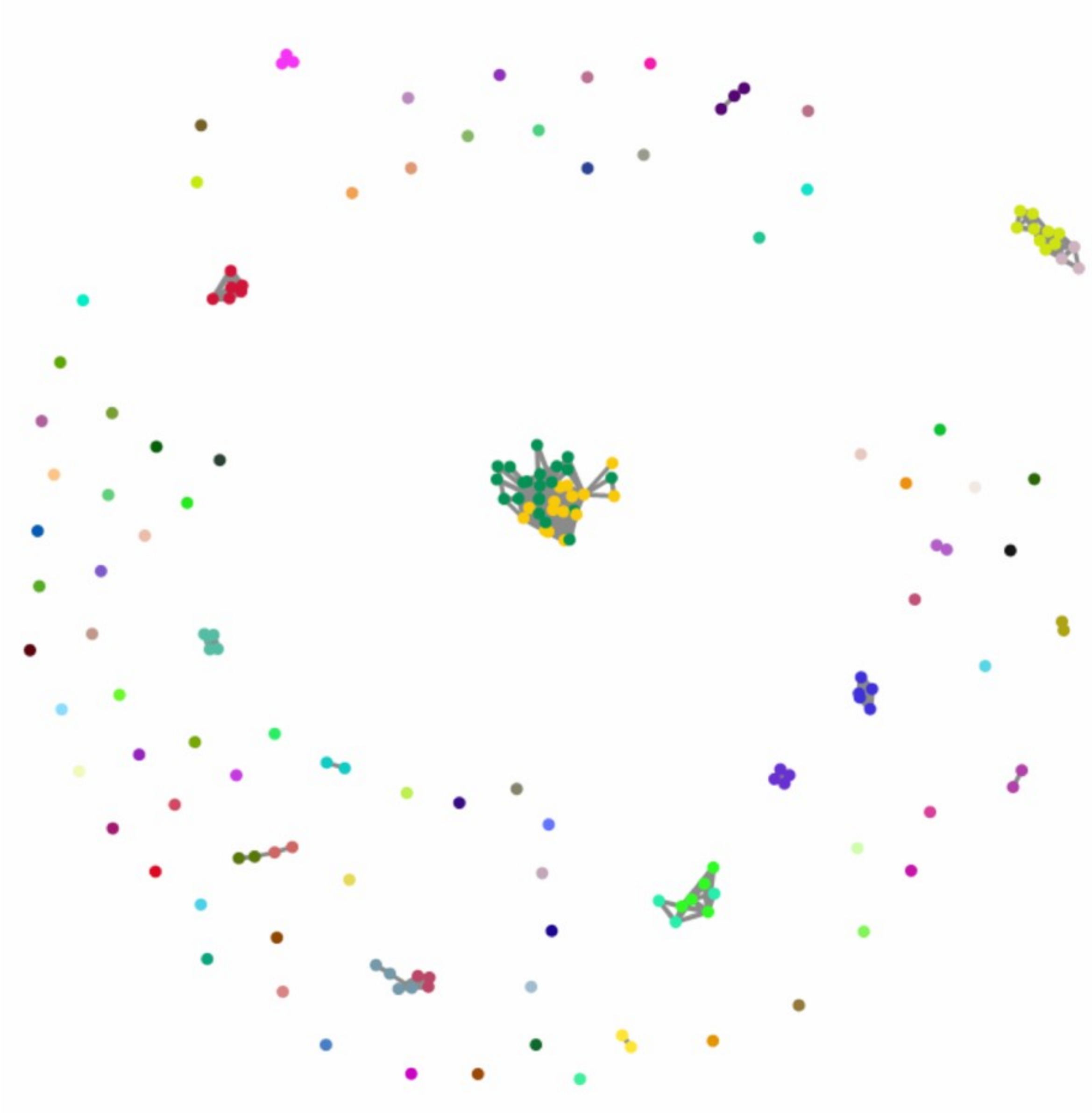

Figure S5: *Micromonospora* - Biosynthetic Gene Cluster Similarity Networks of "PKS-NRPS Hybrids" BGCs

Gene cluster similarity networks of PKS- BGCs generated with BiG-SCAPE from 87 *Micromonospora* genomes. Gene clusters were identified and classified using antiSMASH. Each node represents one sequenced gene cluster. Connected clusters likely encode for similar compounds. To identify already known and characterized BGCs, the dataset from the MIBiG database was added to the network analysis. MIBiG compounds are circled in red.

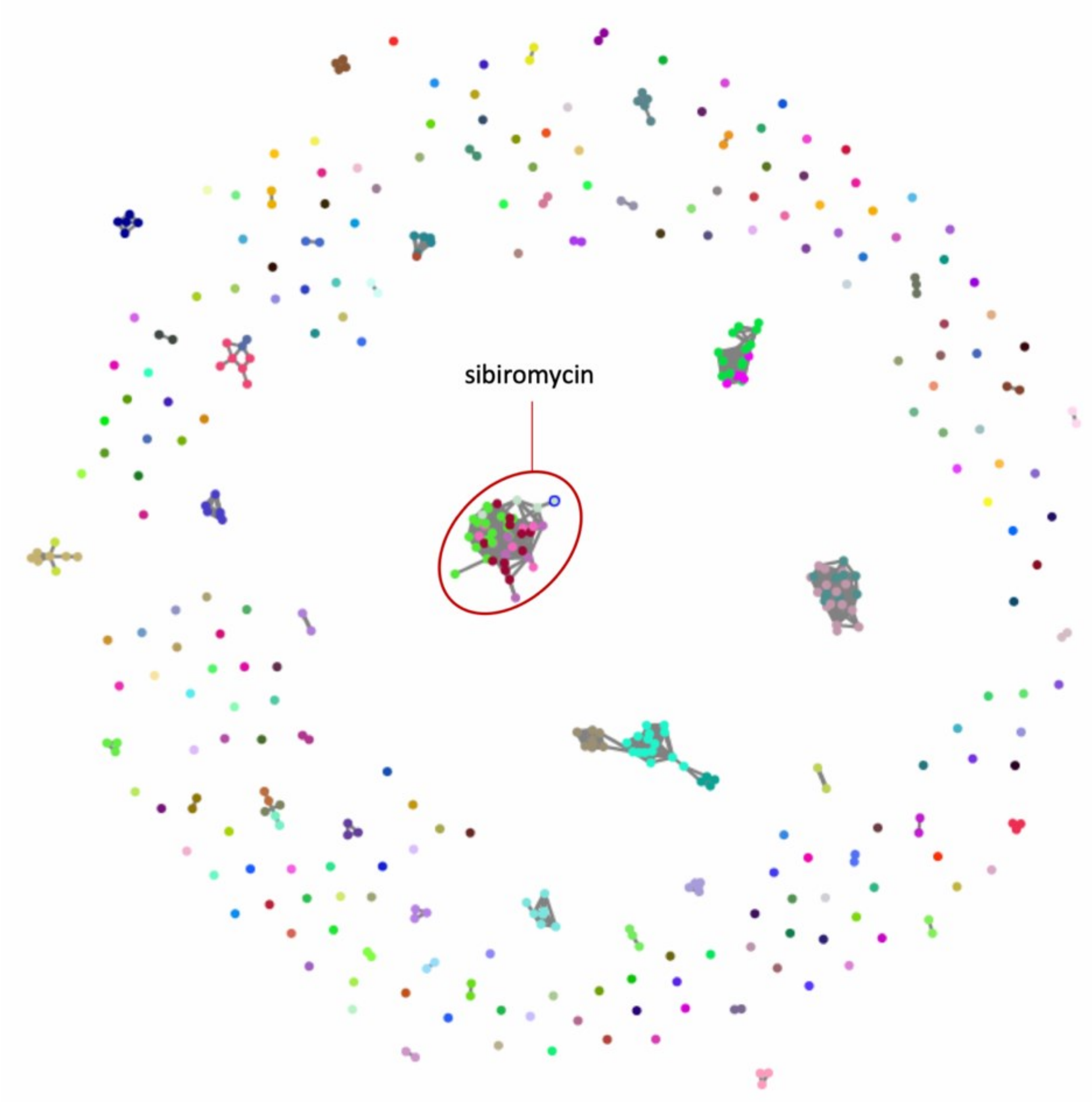

Figure S6: *Micromonospora* - Biosynthetic Gene Cluster Similarity Networks of "NRPS" BGCs

Gene cluster similarity networks of PKS- BGCs generated with BiG-SCAPE from 87 *Micromonospora* genomes. Gene clusters were identified and classified using antiSMASH. Each node represents one sequenced gene cluster. Connected clusters likely encode for similar compounds. To identify already known and characterized BGCs, the dataset from the MIBiG database was added to the network analysis. MIBiG compounds are circled in red.

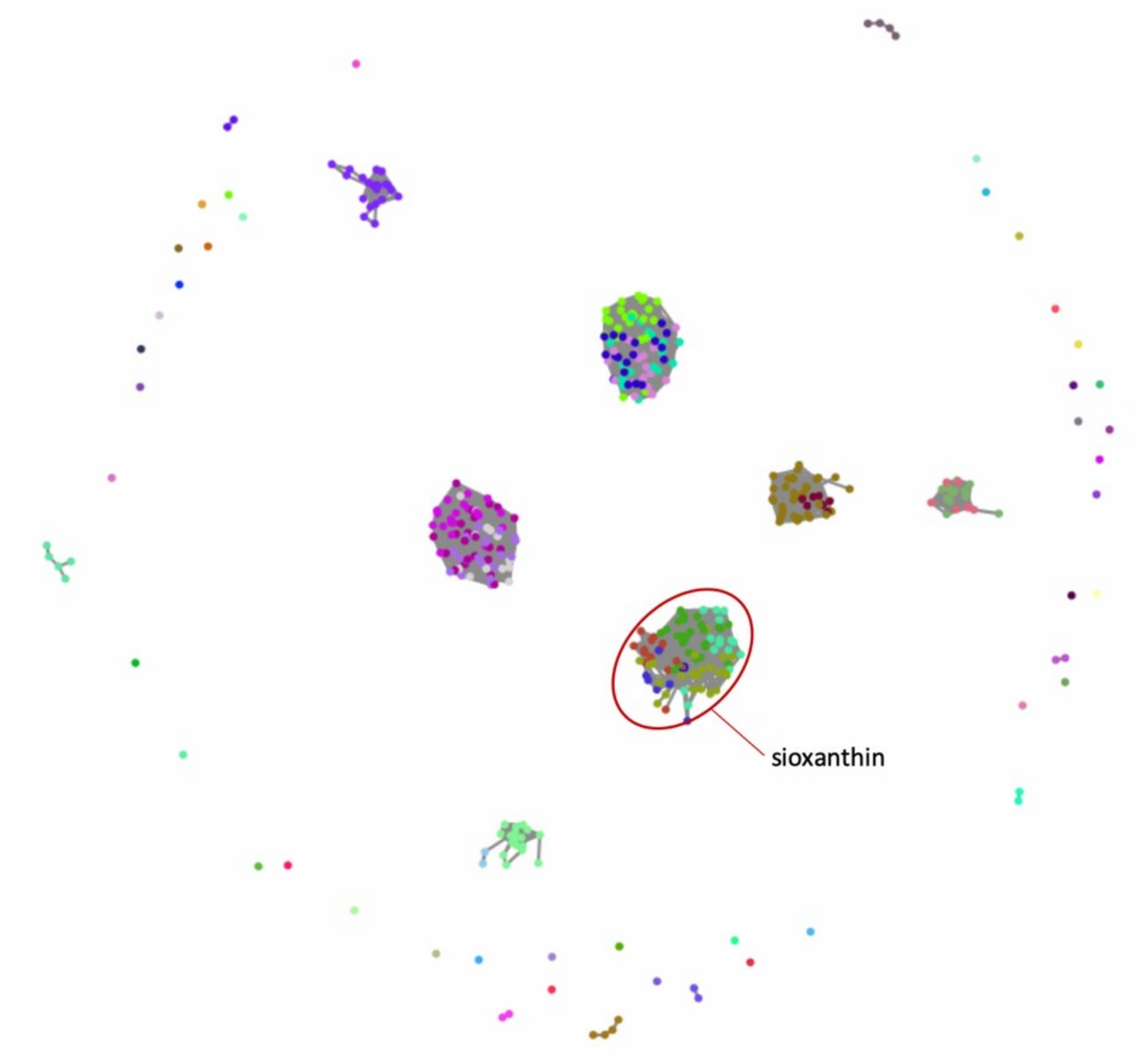

Figure S7: ***Micromonospora*** **- Biosynthetic Gene Cluster Similarity Networks of "Terpene" BGCs**

Gene cluster similarity networks of PKS- BGCs generated with BiG-SCAPE from 87 *Micromonospora* genomes. Gene clusters were identified and classified using antiSMASH. Each node represents one sequenced gene cluster. Connected clusters likely encode for similar compounds. To identify already known and characterized BGCs, the dataset from the MIBiG database was added to the network analysis. MIBiG compounds are circled in red.

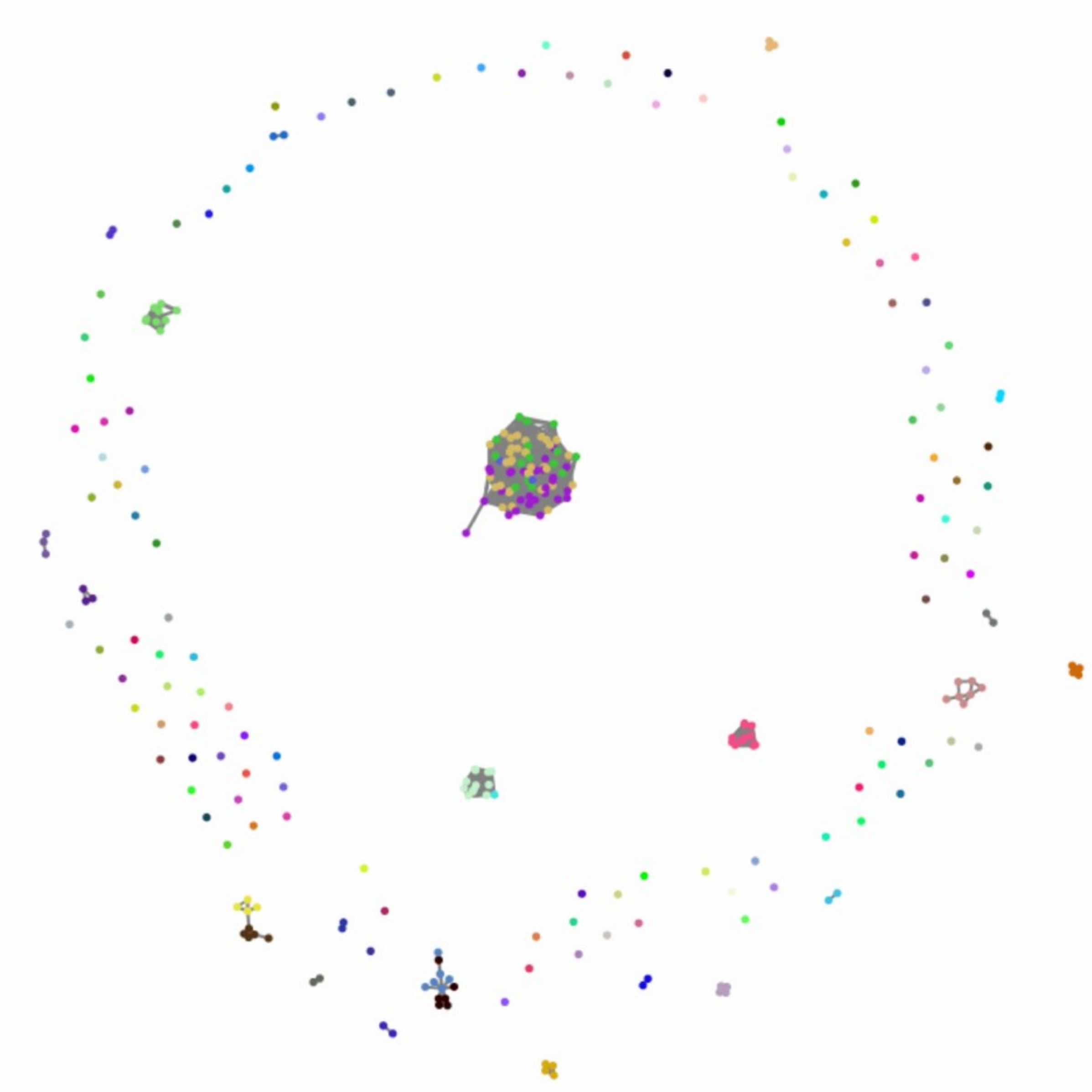

Figure S8: *Micromonospora* - Biosynthetic Gene Cluster Similarity Networks of "RiPPs" BGCs

Gene cluster similarity networks of PKS- BGCs generated with BiG-SCAPE from 87 *Micromonospora* genomes. Gene clusters were identified and classified using antiSMASH. Each node represents one sequenced gene cluster. Connected clusters likely encode for similar compounds. To identify already known and characterized BGCs, the dataset from the MIBiG database was added to the network analysis. MIBiG compounds are circled in red.

Table S1. Natural products derived from *Micromonospora*

Compounds	Class	Microbe	Source	Year	Activity	references
Paromamine(1)	Aminoglycoside	Minor component of the gentamicin complex prod. by *Micromonospora spp*	Soil	1959	Weakly active against Gram-positive bacteria Important intermed. for semisynthetic aminoglycoside	[1]
Gentamicins (2)	Aminoglycoside	*M. echinospora* NRRL 2953 *M. echinospora* NRRL 2985	Soil	1963	Antibacterial	[2]
Antibiotic 460 (3)	Aminoglycoside	*M. chalcea* subsp. flavida NRRL 3222	Soil	1969	Antibacterial MIC (2.5- 7.5 µg/ml) Gram positive bacteria	[3]
6640 (sisomicin) (4)	Aminoglycoside	*M. inyoensis* NRRL 3292.	Soil	1970	Antibacterial activity. MIC values ranged from (0.01-7.5 µg/ml).	[4]
Gentamine C_1 (5)	.Aminoglycoside	*M. purpurea-nigrescens*	Soil	1971	Active mainly against Gram-positive bacteria	[5]
Neomycin B (6)	Aminoglycoside	*M. chalcea* 69-683	-----	1971	Antibacterial activity	[6]
Antibiotic G-418 (7)	Aminoglycoside	*M. echinospora* NRRL 5326	Soil	1974	Antibacterial with MIC values (16-64µg/ml). Antiparasitic activity	[7] [8]

Mutamicins (8)	Aminoglycoside	*M. inyoensis* NRRL 3292	Soil	1974	Antibacterial activity MIC (0.08-3µg/ml).	[9]
Sagamicin (9)<br><br>(XK-62-2)	Aminoglycoside	*M. sagamiensis* subsp. nonreducans ATCC 21803.<br>*M. sagamiensis* ATCC 21826.	Soil	1974	Antibacterial activity MIC (0.001-8.3 µg/ml)	[10]
Verdamicin (10)	Aminoglycoside	*M. grisea* NRRL 3800	Soil	1974	Antibacterial activity.<br>MIC (0.5-8 µg/ml)	[11]
Gentamicin $_{2b}$ (11)	Aminoglycoside	*M. sagamiensis*	Soil	1975	Antibacterial less ototoxic and nephrotoxic than Gentamicin C complex	[12]
Antibiotic G-52 (12)	Aminoglycoside	*M. zionensis* NRRL 5466	Soil	1976	Antibacterial activity for gram positive and gram negative bacteria with IC_{50}(0.01-17.5µg/ml and 0.03-7.5µg/ml respectively	[13]
Antibiotic 66-40B Sisomicin B (13)	Aminoglycoside	*Minor prod. from M. inyoensis*	Soil	1976	Antibacterial	[14]
Antibiotic 66-40D Sisomicin D (14)	Aminoglycoside	*Minor prod. from M. inyoensis*	Soil	1976	Antibacterial	[14]
Destomycin B $C_{21}HC_{21}H_3$	Aminoglycoside	*M. cyaneogranulata*	Soil	1976	Antibacterial	[15]

(15)	$_{39}N_3$ N_3O_{13} O_{13}					
Gentamicin A (16)	Aminoglycoside	*Micromonospora spp*	Soil	1976	Antibacterial	[2]
Gentamicin B,B1 (17,18)	Aminoglycoside	*Micromonospora spp*	Soil	1976	Antibacterial	[8]
Gentamicin C_1,C_{1a} (19,20)	Aminoglycoside	*M. purpurea, M. echinospora, M. sagamiensis, M. scabitana, M.longisporoflavus*	Soil	1976	Antibacterial	[12]
Gentamicin C_{2a} (21)	Aminoglycoside	*M. purpurea and M. sagamiensis*	Soil	1976	NA	[8]
Gentamicin X_2 (22)	Aminoglycoside	*M. purpurea and M. echinospor*	Soil	1976	NA	[8]
Gentoximicin B (23)	Aminoglycoside	*M. purpurea*	Soil	1976	NA	[8]
Fortimicins A and B (24,25)	Aminoglycoside	*M. olivoasterospora* ATCC 21819.<br><br>*Micromonospora species* MK-70	Soil.<br><br>Soil	1976<br><br>1977	Antibacterial against gram positive with with MIC (0.2-10µg/ml). and negative bacteria (0.08-5µg/ml)for fortimicin A.	[16]
Antibiotic I1 (26)	Aminoglycoside	*M.purpurea*	Soil	1977	Antibacterial	[5]
Antibiotic 66-40C (27)	Dimeric aminoglycoside antibiotic	*M.inyoensis*	Soil	1977	NA	[17]
Antibiotic Y 02077Hδ 3''-N-	Aminoglycoside	*M. purpurea and Micromonospora*	Soil	1977	NA	[5]

Demethylgentamicin C_2 (28)		*sp. Y-02077*H				
Garamine (29)	Aminoglycoside	*Micromonospora* cultures. Component of Sisomicin.	Soil	1977	NA	[5]
Gentamicin A_1,A_2,A_3,A_4 (30,31,32,33)	Aminoglycoside	*M. purpurea* and *M. echinospora*	Soil	1977	-------------------	[5]
Gentamine C_1 (34)	Aminoglycoside	*M.purpurea-nigrescens*	Soil	1977	Antibacterial Active against Gram-positive bacteria	[5]
Gentamine C_{1a} (35)	Aminoglycoside	*M. purpurea-nigrescens.*	Soil	1977	Antibacterial Active against gram positive bacteria	[5]
Gentamine C_2 (36)	Aminoglycoside	*M.purpurea-nigrescens.*	Soil	1977	Antibacterial Active against Gram-positive bacteria	[5]
4''-Demethylgentamicin C (37)	Aminoglycoside	*M. purpurea-nigrescens*	Soil	1977	Antibacterial	[5]
4''-Demethylgentamicin C_2 (38)	Aminoglycoside	*M. purpurea-nigrescens*	Soil	1977	Antibacterial	[5]
4'' Demethylgentamicin C_{1a} (39)	Aminoglycoside antibiotic	*M. purpurea-nigrescens.*	Soil	1977	Antibacterial	[5]
6'-Methylgentamicin A (40)	Aminoglycoside	*M. purpurea-nigrescens*	Soil	1977	Antibacterial	[5]
6'-Methylgentamicin A_1 (41)	Aminoglycoside	*M. purpurea-nigrescens*	Soil	1977	Antibacterial	[5]
Antibiotic XK 62-4	Aminoglycoside	*M. sagamiensis*	Soil	1978	Antibacterial	Ger. Pat., 1978, 2

(42)						821 948
3''-N-Demethylsisomicin Antibiotic 66-40G (43)	Aminoglycoside	*M. inyoensis and M. sagamiensis.*	Soil	1978	Antibacterial	[18]
Fortimicin D (44)	Aminoglycoside	*M. olivoasterospora*	Soil	1979	Antibacterial	[19]
Fortimicin KE (45)	Aminoglycoside	*M. olivoasterospora*	Soil	1979	Weak antibacterial	[19]
Antibiotic X 14847(46)	Aminoglycoside	*M. echinospora*	Soil	1980	Antibacterial Active against gram positive bacteria	[20]
Fortimicin E (47)	Aminoglycoside	*M. olivoasterospora*	Soil	1980	Weak antibacterial	[21]
Fortimicin KO_1 Fortimicin AE (48-49)	Aminoglycoside	*M. olivoasterospora*	Soil	1980	Antibactrerial	[22]
Fortimicin AP(50) Fortimicin AM(51)	Aminoglycoside	*M. olivoasterospora*	Soil	1980	Antibactrerial	[23]
Fortimicin AH Fortimicin AI (52,53)	Aminoglycoside	*M. olivoasterospora*	Soil	1980	Antibacterial	[24]
Fortimicin AK (54)	Aminoglycoside	*M. olivoasterospora*	Soil	1980	NA	[24]
Fortimicin AO (55)	Aminoglycoside	*M. olivoasterospora*	Soil	1980	NA	[24]
O-Demethylfortimicin A (56)	Aminoglycoside	*M. olivoasterospora*	Soil	1980	NA	[25]
2'N-Glycylfortimicin KE (57)	Aminoglycoside	*M. olivoasterospora*	soil	1981	NA	[26]
Antibiotic SU1,SU2,SU3,SU4 (58-61)	Aminoglycoside	*M. sagamiensis*	Soil	1982	antibacterial against gentamicin resistant strains	[27]
2-Hydroxysagamicin (62)	Aminoglycoside	*M. sagamiensis* and *M. purpurea*	Soil	1982	Antibacterial	[28]
6'-N-Methylverdamicin (63)	Aminoglycoside	Prod. from Verdamicin by a	Soil	1982	Antibacterial	[29]

		strain of *M. inyoensis* and *M. zionensis* NRRL5466				
5-Deoxygentamicin C_{2b} (64)	Aminoglycoside	*M. purpurea*	Soil	1983	Antibacterial	US Pat., 1983, 4 412 068
Antibiotic FU 10 (65)	Aminoglycoside	*M. olivoasterospora*	Soil	1984	Weak antibacterial	[30]
Fortimicin KK (66)	Aminoglycoside	Micromonospora olivoasterospora	Soil	1984	Antibacterial	[31]
Fortimicin KL1 (67)	Aminoglycoside	Micromonospora olivoasterospora	Soil	1984	Antibacterial	[31]
Vertilmicin (68)	Aminoglycoside	Semisynthetic, prod. by *Micromonospora sp*	Soil	1987	Antibacterial	[32]
Calicheamicins (69)	Aminoglycosidic antibiotic complex. Enediyne antibiotic	*M.echinospora ssp. calichensis* NRRL 15839	Soil	1989	Antineoplastic agent	[33]
Antibiotic Sch 58777 Orthosomycin J (70)	Aminoglycoside	*M. carbonacea var. africana*	Soil	1997	Antibacterial against Staphylococcus aureus	[34]
Fortimicin KR1 (71)	Aminoglycoside	*Micromonospora olivoasterospora*	Sponge	2010	Antibacterial	[35]
Primycin, Debrycin. Ebrimycin (72-74)	Macrolide complex	*M. galeriensis*	Soil	1954	Potent ionophore. Active against Gram-positive bacteria and mycobacteria. Antifungal agent	[36]

Megalomicins A_1,B,C_1,C_2 (75-78)	Macrolides	*M. megalomicea* subsp. megalomicea NRRL 3274 *M. megalomicea* subsp. nigra NRRL 3275	Soil	1969	Antibacterial MIC A (0.075 -1.2 µg/ml) B ((0.005-5 µg/m). C1 (0.003-1.2µg/ml). C2 ((0.0005-0.6 µg/ml). Antiviral C1 Antiparasite (IC_{50} 0.2, 1, 2, 3, and 8 µg/mL) A1.	[37]
Rosamicin (79)	Macrolide	*M. rosaria*	Soil	1972	Antibacterial Gram positive with MIC 0.03-3 µg/ml and Gram negative bacteria with MIC ranged from (0. 3-7.5 µg/ml).	[38]
Antibiotic XK 41B2 (80)	Macrolide	*M. inositola*	Soil	1974	NA	[39]
Juvenimicins A_2,A_3,A_4,B_1,B_3 (81-85)	Macrolides	*M.chalcea var. izumensis*	Soil	1976	Antibacterial Gram +ve MIC (0.01- 100µg/ml) Gram –ve MIC (5->100 µg/ml)	[40] [41]
Antibiotic M 4365G1 (86)	Macrolide	*M. capillata*	Soil	1977	Antibacterial Active against gram positive bacteria	[42]

Repromicin Antibiotic M 4365G2 (87)	Macrolide	*M. capillata and M. rosari*	Soil	1978	Antibacterial Active against gram positive bacteria	[43]
Antlermicin B Antlermicin C (88,89)	Macrolide	*M. chalcea-kazunoensis* sp. T-90.	Soil	1980	Antibacterial Antitumor	[44]
Mycinamycin I,II.III.IV,V (90-94)	Macrolide	*M. grisseorubida A11725*	Soil	1980	Active against gram positive bacteria, Haemophilus influenzae and mycoplasmas (MIC 0.1 – 3.12 µg/mL)	[45]
Protylonolide (95)	Macrolide	*M. rosaria*	Soil	1980	Biosynth. precursor to Tylonolide.	[46]
20-Deoxorosaranolide (96)	Macrolide	*M. rosaria*	Soil	1982	Prob. intermed. in biosynth. of Rosamicin	[47]
Lipiarmycin A_3 (97)	Macrolide	*M. echinospora ssp. armeniaca*	Soil	1983	Antibacterial	[48]
23-Hydroxyprotylonolide (98)	Macrolide	*Micromonospora sp.* YS-02930K	Soil	1983	NA	[49]
19,23-Dihydroxyprotylonolide (99)	Macrolide	*Micromonospora sp. YS-02930*K	Soil	1983	NA	[49]
Neorustmicin A (100)	Macrolide	*M. chalcea* 1302-AV	Soil	1985	Antifungal , MIC (0.2-0.4µg/ml).	[50]

Rustmicin (101)	Macrolide	*M. narashinoensis* 980-MC.	Soil	1985	Antifungal MIC (0.8-1µg/ml).	50
Galbonolide A (102)	Macrolide	*M.narasinhoensis and M. chalcea*	Soil	1985	Antifungal	51
Clostomicins A, B_1, B_2, C, D (103-107)	Macrolide	*M. echinospora* subsp. *armeniaca* KMR-593	Soil	1986	Antibacterial Diameter of inhibition zone (mm) (10.2 – 36.8).	52
Neorustmicin B,C,D (108-110)	Macrolide	*M. chalcea* 1302-AV	Soil	1986	Antifungal Neorustmicin B 1.0 µg/ml While, neorustmicins C and D 4 and 5 µg/ml, respectively	. 53
Lipiarmycin B_3 (111)	Macrolide	*M. echinospora*	Soil	1988	Antibacterial Active against gram positive bacteria	54
Izenamicin B2 (Glycoside , aglycon) A_1-A_4 B_1-B_4 (112-115)	Macrolide	*Micromonos pora sp* S-02930K.	Soil	1989	Antibacterial	55

Antibiotic 6108A_1 (116)	Macrolide	*M. fastidiosus*	Soil	1990	Antibacterial	[56]
Antibiotic 6108 A_1, B (117,118)	Macrolide	Micromonospora strain BA06108	Soil	1990	Antibacterial Gram-positive with MIC (0.1-0.39 µg/ml)and some Gram-negative MIC (0.1-50µg/ml)	[57]
Antibiotic 6108C (119) Antibiotic 6108D (120)	Macrolide antibiotic (unusual Tylosin-type)	*M. Pora fastidiosa*	Soil	1990	Antibacterial	[56] [58]
Rosamicin;6-Hydroxy (121)	Macrolide	*M. rosaria*	Soil	1990	NA	[57]
Mycinamycin X, XI (122,123)	Macrolide	*M. griseorubida*	Soil	1991	Antibacterial mainly against gram positive bacteria	[59]
Mycinamycin IX, XII, XIII, XIV, XV, XVI, XVII, XVIII (124-131)	Macrolide	*M. griseorubida*	Soil	1991	Active against gram positive bacteria with MIC value ranged from (0.05- 12.5µg/ml).	[59]
AC6H (132)	Macrolide Spirotetronate glycoside.	*M. carbonacea* subsp. carbonacea K55-AC6	Soil	1993	Anticancer IC 50 (6.25-25 µg/ml)	[60]
Quinolidomicins A_1, A_2, and B_1 (133-135)	Polyene macrolides	*Micromonospora sp.* JY16 - FERM BP-3940	Soil	1993	Antitumor IC_{50} 327nM/ml).	[61]

19-Decarbonyltylonolide (136)	Macrolide	*Micromonospora sp.* YS 02930k	Soil	1994	NA	[62]
16-Hydroxyprotylonolide (137)	Macrolide	*Micromonospora sp.* YS-02930K	Soil	1994	NA	(Yasumuro et al., 1994
19-Hydroxyprotylonolide (138)						
Royamicin A(139)	Macrolide	*M. roseopurpurea M90*	Soil	1994	Antibacterial Antifungal	[63]
Pyrrolosporin A (140)	Macrolide	*Micromonospora sp.* ATCC 53791	Soil	1996	Antibacterial Gram positive MIC (0.5 - 4 µg/ml) Gram negative MIC (63-125µg/ml) Antitumor	[64] [65]
Galbonolide B, 21-hydroxy (141)	Macrolide	*Micromonospora sp.* culture MA	----------	1998	Moderate antifungal activity.	[66]
Rustmicin, 21-hydroxy (142)	Macrolide	Micromonospora sp. culture MA	----------	1998	Antifungal activity less than rustamicin	[66]
Antibiotic IB 96212 (143)	Macrolide	*Micromonospora sp.*	Marine	2000	Cytotoxic	[67]
Sch 351448 (144)	Macrolide	*Micromonospora* sp	Soil	2000	A novel ionophoric compound and is a weak activator of low density	[68]

					lipoprotein receptor (LDL-R) promoter with an IC_{50} 25 μM	
Micromonospolide A,B,C (145-147)	Macrolides	*Micromonospora sp. Nov*.		2001	Specific inhibitor of starfish embryogenesis. Micromonospolide A,B,C MIC of 0.01, 0.011, and 1.6 μg/mL, respectively.	69
Bafilomycin R 176502 Antibiotic R 176502 (148)	Macrolide antibiotic	*Micromonospora sp.*	River sediment	2003	Cytotoxic	70
Micromonosporin A (149)	Macrolide	*Micromonospora sp*. (strain TT1-11).	Acidic peat swamp forest.	2004	NA	71
IZI (150)	Macrolide	*M. rosara* TPMA0001	---------	2009	NA	72
IZII,IZIII (151,152)	Macrolides	*M. rosara* TPMA0001	-----------	2010	NA	73
levantilide A and B (153,154)	Macrolides	*Micromonospora* strain M71-A77	Marine	2011	Anticancer against gastric tumor cells GXF 251L (IC_{50} 40.9 μM), lung tumor cells LXFL 529L (IC_{50} 39.4 μM), mammary tumor cells MAXF 401NL (IC_{50} 28.3	74

					μM), melanoma tumor cells MEXF 462NL (IC_{50} 48.6 μM), pancreas tumor cells PAXF 1657L (IC_{50} 20.7 μM) and renal tumor cells RXF 486L (IC_{50} 52.4 μM).	
Juvenimicin C, 5-O-alpha-L-rhamnosyltylactone (155)	Macrolide	*Micromonospora sp.*	Marine	2013	Antioxidant enhanced QR1 enzyme activity and glutathione levels by two-fold with CD values of 10.1 and 27.7 μM, respectively. QR1 (quinon reductase 1) Anticancer	[75]
Levantilide C (156)	Macrolide	Micromonospora sp. FIM07-0019	Marine	2013	Anticancer Against HL-60 (IC_{50} 32.5 μM), MDA-MB-231 (IC_{50} 26.8 μM), SW620 (IC50 16.4 μM) SMMC7721 (IC_{50} 39.9 μM)	[76]

Micromonolactam (157)	Macrolide	*Micromonospora sp*	Marine	2013	NA	77
Neaumycin B (158)	Macrolide	Micromonospora sp. (strain CNY-010)	Surface of the brown alga Stypopodium zonale,	2018	Potent Inhibitor of Glioblastoma IC_{50} (1µM).	78
Tetrocarcin A Antlermicin A (159,160)	Tetrocarcin	*M. chalcea* subsp. *kazunoensis*	Soil	1980	Antibacterial MIC (0.015 µg/ml) Antitumor Antimalarial	79
Tetrocarcin complex A, B, C (161-163)	Tetrocarcin Spirotetronate glycosides	*M. chalcea* KY11091	Soil	1980	Antitumor	80
Tetrocarcin G,H,K,L (164-167)	Tetrocarcin Spirotetronate glycoside	*M. chalcea* KY11091	Soil	1980	Antibacterial	80
Tetronolide Antibiotic F2 (168)	Aglycon of tetrocarcin A	*M. chalcea*	Soil	1980	Antibacterial Antitumor	80
Tetrocarcin E1 (A) Tetrocarcin E2 (A) Tetrocarcin F1 (A) Tetrocarcin M Tetrocarcin J Tetrocarcin I (A)	Spirotetronate glycoside	*M. chalcea*	Soil	1982	Antibacterial MIC (3-150µg/ml).	81

Tetrocarcin F (A)<br>Tetrocarcin C (A)<br>Tetrocarcin D (A)<br>Tetrocarcin L<br>Tetrocarcin K Tetrocarcin B<br>(169-180)						
Arisostatin A&B<br>(181,182)	New analogs of tetrocarcin A	*Micromonospora* sp. TP-A0316	Sea water sample	2000	Antibacterial MIC (0.39-25μM. Antitumor IC50 (0.059- 0.26 μM)	[82]
Tetrocarcin P<br>(183)	Tetrocarcin<br><br>Spirotetronate glycoside.	*M. harpali* SCSIO GJ089.	Sediment sample. Marine	2017	Antibacterial MIC (1 -2μg/ml) against Bacillus.	[83]
22-dehydroxymethyl-kijanolide<br>(184)	Spirotetronate glycoside.	*M. harpali* SCSIO GJ089.	Sediment sample. Marine	2017	NA	[83]
8-hydroxy-22-dehydroxymethyl-kijanolide<br>(185)	Spirotetronate glycoside.	*M. harpali* SCSIO GJ089.	Sediment sample Marine	2017	NA	[83]
Microsporanates A-F<br>(186-191)	Spirotetronate glycoside.	*M. harpali* SCSIO GJ089.	Sediment sample Marine	2017	Antibacterial activity	[83]
Tetrocarcin N, H,Q<br>(192-194)	Tetrocarcin<br><br>Spirotetronate glycoside	*M. carbonacea* LS276	sponge *Gelliodes carnosa,*	2018	Antibacterial activity against *Bacillus subitlis* (MIC) value of 12.5 μM.	[83]

Actinomycins (195)	Polypeptide	*Micromonospora sp.* 608	Soil	1951	Anticancer	[84]
Microcins A and B (196 -197)	Peptides	*M. fuscus*	Soil	1952	---------------	(Taira and Fugii ., 1952)
Bottromycin (198)	Cyclic peptide	*M. chalcea*	Soil	1966	NA	[85]
Antibiotic SF 1919 (199)	Peptide.	*Micromonospora sp.* SF-1919,	Soil	1977	Antibacterial	Japan. Pat., 1977, 77 136 995
Antibiotic 68-1147 (200)	Thiazole-peptide	*M. arborensis NRRL8041*	Soil	1978	Antibacterial	[86]
Sch 18640 (201)	Peptide	*M. arborensis NRRL8041*	Soil	1978	Antibacterial	. [86]
Epideoxynegamycin (202)	peptide	*Micromonospora sp*	Soil	1979	NA	[87]
Antibiotic PA 4046-I (203)	peptide	*M. miyakonensis* PA4046	Soil	1981	NA	[88]
N-(2,6-diamino-6-hydroxymethylpimelyl) -L-alanine (204)	Dipeptide	*M. chalcea* PA-3534	Soil	1981	Antibacterial	[89]
Antibiotic PA 3534J (205)	Dipeptide	*M. chalcea* PA-3534	Soil	1981	Active against E. coli.	[89]
Antibiotic M 9026 (206)	Peptide antibiotic complex	*Micromonospora sp.*	Soil	1987	Antibacterial Anticancer	[90]
Antibiotic S 54832A (207)	Depsipeptide antibiotics	*M. globosa*	Soil	1984	Active against Gram-positive bacteria, mycoplasms and Neisseriae sp	[91]
Antibiotic S 54832A-I (208)	Depsipeptide antibiotics	*M. auratinigra*	Soil	1984	No activity	[91]
Chloropolysporin B Chloropolysporin C	Glyco peptide	*Micromonospora sp.*	Soil	1987	Antibacterial Animal growth	[92]

(209,210)					promoter.	
Sch 37137 (211)	Dipeptides	*Micromonospora sp.* SCC 1792	Soil	1988	Antifungal activity against Candida sp. (MICs >12 μg/ml) and dermatophytes (MICs >0.8 μg/ml).	93
Korkormicins A-G) (212 – 218)	Depsipeptide	*Micromonospora sp.* C39500	Soil	1995	Antibacterial MIC (0.13- 0.5 μg/ml). Anticancer (in vivo 0.05-0.20 mg/ kg(IP) .	94
Rakicidin A (219)	Cyclic lipopeptide	*Micromonospora sp.* R385-2a	Soil	1995	Cytotoxic IC 50 (40ng/ml).	95
Rakicidin B Antibiotic FW 523-3 (220)	Cyclic lipopeptide	*M. chalcea* and a *Micromonospora sp.*	Marine	1995	Anticancer, Induces apoptosis. Immunosuppressa nt. IC_{50} (200ng/ml).	95
Thiocoraline (221)	Thiodepsipeptide	*Micromonospora sp.* ACM2-092	Marine ,Soft coral and mollusk.	1997	Cytotoxic activity IC50 (0.002μg/ml). Antibacterial MIC (0.03-0.05 μg/ml)	96
Antibiotic Sch 40832 (222)	Peptide	*M. carbonaceae* var. *africana*	Soil	1998	Antibacterial	97
Antibiotic Sch 49088 (223)	Oligosaccharide	*M. carbonaceae*	Soil	1998	NA	98
Actinomycin Z_1-Z_5	Chromopeptide	*M. floridensis*	Soil	2000	Antibacterial , B.	99

(224-228)					subtilis. MIC 12.5, 0.20, 0.78 μg/ml. Anticancer IC_{50} (0.28-50 μg/ml).	
Telomycin (229)	Macrocyclic peptide lactone	*M. schwarzwaldensis*	Soil	2013	Antibacterial activity	[100]
Cyclo-(Pro-Trp) (230)	Peptide	*Micromonospora* sp. (strain G044)	sponge *Tethya aurantium*	2017	Antibacterial against E-coli MIC 128μg/ml.	[101]
Cyclo-(Pro-Met) (231)	Peptide	*Micromonospora* sp. (strain G044)	sponge Tethya aurantium	2017	NA	[101]
Cyclo-(Pro-Val) (232)						
Uridine (233)	Peptide	*Micromonospora* sp. (strain G044)	sponge Tethya aurantium	2017	NA	[101]
Rakicidins G, H, I (234-237)	Cyclic depsipeptides	*M. chalcea* FIM 02-523	Marine	2018	Cytotoxic IC 50 (0.00783-0.0207 μg/ml))hypoxia IC_{50} (0.148-0.188μg/ml). Antibacterial MIC (0.125-8μg/ml)	[102]
Rakicidin E (238)	Cyclic depsipeptide	*M. chalcea* FIM 02-523	Marine	2018	Cytotoxic Antibacterial MIC (2-32 μg/ml)	[102]
Izumenolide (239)	Lactone	*M. chalcea* subsp. *izumensis*	Soil	1980	Antibacterial	[103]

		SC 11133				
Dotriacolide (240)	Lactone	*M. echinospora* MG299-fF35	Soil	1981	Antibacterial	[104]
3, 4-Dihydrodotriacolide (241)	Lactone	*M. echinospora*	Soil	1981	NA	[104]
Antascomicins A,B,C,D,E (242-246)	Macrocyclic lactones	*Micromonospora sp.* DSM 8429	Soil	1996	Antagonize the immunosuppressiv e activity of FK506 and rapamycin (FKBP12 binding molecules) (IC_{50} 0.7 nM)	[105]
Cymbimicin A and B (247,248)	Lactone	*Micromonospora sp.* DSM 8594	Soil	1997	Immuno-suppressive.	[105]
Antibiotic PA 2046 (249)	Pyranonaphthoquinon e	*M. nakanoshimensis*	Soil	1981	Antitumor	Japan. Pat., 1981, 81 73 096
M-92, M-92 BN-3, M-92 VA-2, M-92 BA-4, M-92 BA-5, M-92 BN-1, M-92 BN-2 (250-256)	Naphthoquinone	*M. verruculosa* M-92	Soil	1982	Antibacterial activity Anticancer activity	[106]
Crisamicin A (257)	Naphthoquinone	*M. purpureochromogen es* subsp. *halotolerans* RV-79-9-101	Soil	1986	Antibacterial activity MIC (0.2-10µg/ml). Anticancer activity	[107]
Crisamicin C (258)	Naphthoquinone	*M. purpureochromogen es*	Soil	1988	Antibacterial MIC (0.125-0.25µg/ml).	[108]

Crisamicin C (Epoxide) (259)	Naphthoquinone	*M. purpureochromogenes*	Soil	1988	NA	[108]
Antibiotic A 35566B (260)	Naphthoquinone	*Micromonospora sp*. SANK 6039	Soil	1995	Cytotoxic	Japan. Pat., 1995, 95 316 091
Antibiotic A 35566A (4'-Ketone) (261)	Naphthoquinone	*Micromonospora sp*. SANK 6039	Soil	1995	Cytotoxic	Japan. Pat., 1995, 95 316 091
9-Hydroxycrisamicin A (262)	Naphthoquinone	*Micromonospora sp*. SA246	Soil	1997	Antibacterial ,cytotoxic	[109]
1-Hydroxycrisamicin A (263)	Naphthoquinone	*Micromonospora sp.* SA246	Soil	1997	Antibacterial , MIC (0.78-3.12 µg/ml) cytotoxic	[109]
7-Methoxy-2-propyl-5,12-naphthacenedione (264)	Naphthoquinone	*Micromonospora sp.* JN79761	Marine	2012	NA	[110]
1,2,3,4-Tetrahydro-2-hydroxy-7-methoxy-2-propyl-5,12-naphthacenedione (265)	Naphthoquinone	*Micromonospora sp.* JN79761	Marine	2012	NA	[110]
K 259-2 (266)	Quinone	*M. Olivasterospora*	Soil	1987	Inhibitor of Ca2+ and cyclic nucleotide phosphodiesterase. Vasodilator.	[111]
Citreamicin ξ<br>Citreamicin β<br>Citreamicin γ<br>Citreamicin η<br>(267-270)	Quinone	*M. citrea*	Soil	1990	Feed additive	[112]

Antibiotic GTRI-BB (crysamicin analog) (271)	Quinone	*Micromonospora sp.* SA-24	Soil	2002	Cytotoxic GI_{50} (0.08-0.31μg/ml).	[113]
Streptonigrin 7-(1-methyl-2 oxopropyl)streptonigrin (272)	Quinone	*Micromonospora sp.* IM 2670	Soil	2002	Cytotoxic activity	[114]
Kosinostatin (273)	Quinocycline	*Micromonospora sp.* TPA0468	Marine	2002	NA	[115]
Fluostatins C-F (274-277)	Quinone	*M. rosaria* SCSIO N160	Marine	2012	NA	[116]
Fluostatins I−K (278-280)	Quinone	*M. rosaria* SCSIO N160	Marine	2012	NA	[116]
Phenanthroviridone (281)	Quinone	*M. rosaria* SCSIO N160	Marine	2012	Antibacterial Staphylococcus aureus MIC 1.0 μg/mL Antitumor IC_{50} (0.09 ± 0.04 - 2.18 ± 0.01μM)	[116]
Lagumycin B (282), Dehydrorabelomycin (283), WS-5995 A (284)	Angucycline	Micromonospora sp.	Marine	2015	Cytotoxic	117
Cervinomycin A_1 (285)	Xanthone	*Micromonospora sp. M39*	Soil	2004	Antibacterial	[118]
Dynemicin A (286)	Anthraquinone (Enediyne antibiotic)	*M. chersina* ATCC 53710	Soil	1989	Antibacterial activity Anticancer activity	[119]
Deoxydynemicin A (287)	Anthraquinone	*M. globosa* FERM P-10651	Soil	1990	Antibacterial activity	[120]

Dynemicins L, M, and N (288-290)	Anthraquinones	*M. chersina* M 965-1	Soil	1991	Antibacterial , cytotoxic less than dynemicin A	[121]
Dynemicins O, P, and Q (291-293)	Anthraquinones	*M. chersina* M 965-1	Soil	1991	Antibacterial cytotoxic	[122]
Endynamicin A and B (294-295)	Anthrquinones	*M. globosa*	Soil	1991	Antineoplastic	Japan. Pat., 1991, 91 63 281
Dynemicin C (296)	Enediyne antibiotic	*M. chersina*	Soil	1992	NA	Eur. Pat., 1992, 484 856
Lupinacidins A, B (297,298)	Anthraquinone	*M. lupine* Lupac 08	Root nodules of Lupinus Angustifolius	2007	Anticancer	[123]
2-Ethyl-1,8-dihydroxy-3-Methylanthraquinone (299)	Anthraquinone	*M. rhodorangea*	Marine	2009	NA	[124]
3,8-Dihydroxy-1-propylanthraquinone (300)	Anthraquinone	*M. rhodorange*	Marine	2009	NA	[124]
3,8-Dihydroxy-1-propylanthraquinone-2-carboxylic acid; 3-Me ether (301)	Anthraquinone	*M. rhodorange*	Marine	2009	NA	[124]
Lupinacidin C (302)	Anthraquinone	*M. lupini Lupac 08*	Root nodules of Lupinus Angustifoli	2011	Anticancer	[125]

			us			
Homo-ε-rhodomycinone (303)	Anthraquinone	*Micromonospora sp. JN797618*	Marine	2012	Cytotoxic	[126]
Rabelomycin (304)	Anthraquinone	*M. rosaria* SCSIO N160	Marine	2012	Antibacterial Staphylococcus aureus MIC 0.25 µg/ml Antitumor IC50 (4.28 ± 0.08-9.91 ± 0.08).	[116]
Nocardorubin (305)	Anthracycline	*M. narashino*	Soil	1954	Antibacterial agent	[127]
Doxorubicin, 11-deoxy Daunorubicin, 11-deoxy-13-dihydro Daunorubicin, 11-deoxy-13-deoxo (306-308)	Anthracycline glycosides	*Micromonospora spp*	Soil	1980	Antibacterial Cytotoxic	[128]
Micromonosporin C Micromonosporin B (309,310)	Anthracycline	*Micromonospora sp. ATCC 10026*	Soil	1987	Antitumor Antibacterial	[129]
Spartanamicins A and B (311,312)	Anthracycline	*Micromonospora sp.* ATCC 53803	Soil	1992	Antifungal MIC (0.2-1µglml).	[130]
Cororubicin (313)	Anthracycline	*Micromonospora sp.* JY16	Soil	1994	Antitumor , cytotoxic	[131]
Bravomicin A ,B,C,D,E and F (314-319)	Anthracycline	*M. polytrota* ATCC 202091	Soil	1999	Antibacterial	US Pat., 1999, 5 994 543

Kosinostatin (320)	Anthracycline	*Micromonospora sp. TP-A0468*	Marine	2002	NA	[115]
Micromonomycin (321)	Anthracycline	*Micromonospora sp.*	Soil	2004	Antibacterial activity<br>Antifungal activity	[132]
Keyicin(322)	Anthracycline	*Micromonospora sp.*	Marine	2017	Antibacterial	[133]
Galtamycin B (323)	Anthracycline	*Micromonospora sp. Tü* 6368	Soil	2005	Cytostatic activity < 1µg/ml.	[134]
Anthracyclinones (324)	Anthracyclinones	*Micromonospora sp.*	Tunicate Eudistoma vannamei	2012	NA	[126]
Echinosporamicin (325)	Echinosporamicin	*M. echinospora* subsp. *echinospora* LL-P17	Soil	2004	Antibacterial Against MRSA MIC < 0.12 µg/ml.	[135]
TLN-05220<br>TLN-05223<br>(326-327)	Echinosporamicin -type antibiotics	*M. echinospora* subsp. *challisensis NRRL* 12255	Soil	2009	Antitumour antibacterial	[136]
Everninomicins (328)	Oligosaccharides	*M. carbonacea* NRRL 2972<br>*M. carbonacea* subsp. *aurantiaca* NRRL 2997<br>*M. carbonacea var. africana* ATCC39149	Soil	1964	Antibacterial	[137]
Orthosomycin B<br>Orthosomycin C<br>Orthosomycin D<br>(329-331)	Oligosaccharide	Micromonospora carbonacea var. Africana	Soil	1997	Show activity against Staphylococcus aureus	Pat. Coop. Treaty (WIPO), 1997, 97 13 777

SCH-27899 (Ziracin) (332)	Oligosaccharide	*M. carbonacea*	Soil	1999	Antibacterial activity	[138]
Antibiotic Sch 58761 Orthosomycin A (333)	Oligosaccharide	*Micromonospora carbonaceae*	Soil	2000	Active against multidrug-resistant bacteria	[139]
Antibiotic Sch 58773 Orthosomycin G (334)	Oligosaccharide	*M. carbonacea var. africana*	Soil	2002	NA	[34]
Antibiotic Sch 58771 Orthosomycin F (335)	Oligosaccharide	*M. carbonacea* var. *africana*	Soil	2002	Active against S. aureus	[34]
Antibiotic Sch 58769 (336)	Oligosaccharide	*M.carbonacea* var. *africana*	Soil	2002	Active against S. aureus	[34]
Garosamine (L-form) (337)	Sugar	Sugar component of Gentamicin C1a and Gentamicin C1, antibiotic complexes from fermentations of *Micromonospora.*	----	1977	NA	[5]
Trehazolin (338)	Pseudodisaccharide	*Micromonospora sp.* SANK 62390	Soil	1991	Trehalase glycosidase inhibitor	[140]
Sibanomicin Antibiotic SF 2364 (339)	Pyrrole benzodiazepines	*Micromonospora sp.* SF2364	Soil	1988	Anticancer Antibacterial Gram + ve (MICs12.5-100 µg/ml) Gram –ve (50 -	[141]

					>100).	
BU-4664L (340)	Dibenzazepines	*Micromonospora sp.* ATCC 55378	Soil	1996	Anti-inflammatory anti-tumor	[142]
Neihumicin (341)	Pyrazines	M. neihuenis NH3-1 Wu	Soil	1988	Cytotoxic activity. Antifungal activity. IC 50 (0.49 µg/ml)	[143]
LL-E19085 alpha Citreamicin α (342)	Oxazole Quinone	*M. citrea* NRRL 18351	Soil	1989	Antibacterial MIC < 0.12 µg/ml.	[144]
Citreamicins (343)	Oxazole Quinone	*M. citrea* NRRL 18351	Soil	1990	Antibacterial activity MIC <0.015µg/ml.	[112]
Trehalamine (344)	Oxazoles	*Micromonospora sp.* SANK 62390	Soil	1993	Inhibit rat intestinal sucrase	[145]
5-Chloro-6-methoxy-1-methylisatin (345)	Indole	Metab. of *M.carbonaceae*	Soil	1967	NA	[146]
5'-hydroxystaurosporine (346)	Indol carbazole alkaloid	*Micromonospora sp.*L-31-CLCO-002	Sponge	2000	Cytotoxic activity IC50 (0.002-0.02 µg/ml).	[147]
4'-N-methyl-5'hydroxystaurosporine (347)	Indol carbazole alkaloid	*Micromonospora sp.*L-31-CLCO-002	Sponge	2000	Cytotoxic activity IC50 (0.002-0.04 µg/ml).	[147]
Skatole-2-carboxylic acid<br><br>3-Methyl-1H-indole-2-carboxylic acid (348)	Indole	*Micromonospora sp.* P1068.		2005	NA	[148]
5-Chloro-1H-indole-3-	Indole	*Micromonospora*	Marine	2013	NA	[76]

carboxylic acid<br>(349)		*sp.* FIM07-<br>0019				
3-Hydroxymethyl-β-carboline (350),<br>3-Methyl-β-carboline (351),<br>β-Carboline (352)	β-carboline	Micromonospora sp. M2DG17	Marine	2011	Cytoxix	149
Antibiotic MS 444<br>Antibiotic BE 34776<br>(353)	Furan	*Micromonospora ssp*		1995	Antitumour, vasodilator, inhibitor of myosin light chain kinase	[150]
Antibiotic SB 219383<br>(354)	Furan	*Micromonospora sp* NCIMB<br>40684	Soil	2000	Tyrosyl tRNA synthetase inhibitor.<br>Antibacterial activity<br>MIC (0.32-0.64 μg/ml).	[151]
3-(4-Hydroxyphenyl)-N-methylpropanamide<br>(355)	Amide	*Micromonospora sp. P1068*		2005	NA	[148]
Lomaiviticins<br>A and B<br>(356,357)	Dimeric diazobenzofluorene glycosides	*M. lomaivitiensis* LL-37I366	Ascidian Polysyncratonlithostrotum	2001	Antitumor<br>Antibacterial	[152]
Sch 725418<br>(358)	Diketopiperazine	*Micromonospora sp.*	-------------	2004	Antifungal<br>MIC (32μg/ml).	[153]
Diazepinomicin<br>(359)	Natural dibenzodiazepine	*Micromonospora sp.* DPJ12<br><br>*Micromonospora sp.* RV115	Ascidian *Didemnum Proliferum*<br><br>Sponge *Aplysina aerophoba*	2004	Anticancer activity IC50 = 72.4 ± 5.3 μM)<br>Anti-inflammatory<br>Antiparasitic activity	[154]

Halomicin D (360)	Ansamycin	*M. halophytica*	Soil	1967	Antibacterial Antifungal	[155]
Rifamycins (361)	Ansamysins	*M. lacustris* ATCC 21975	Soil	1977	Antibacterial activity	[156]
3-(Methylthio)rifamycin (362)	Ansamycin	*M. lacustris*	Soil	1977	Antibacterial Active against gram positive bacteria	[156]
3-(Methylthio)rifamycin; 16,17,18,19,28,29-Hexahydro (363)	Ansamycin	*M. lacustris*	Soil	1977	NA	[156]
3-(Methylthio)rifamycin S (364)	Ansamycin	*M. lacustris*	Soil	1977	Antibacterial Active against gram positive bacteria	[156]
3-(Methylthio)rifamycin; 1,4-Quinone, 16,17,18,19,28,29-hexahydro (365)	Ansamycin	*M. lacustris*	Soil	1977	NA	[156]
Antibiotic CP 43038 Antibiotic CP 42752 Antibiotic CP 43139 (366-368)	Ansamycin	*M. saitamica.*	Soil	1977	NA	US Pat., 1977, 4 032 631
Halomicins A,B,C (369-371)	Ansamysins	*M. halophytica* subsp. *halophytica* NRRL 2998<br><br>*M. halophytica* subsp. *nigra* NRRL 3097	Salt pool	1977	Antibacterial Antifungal	[157]
Rifamycin S (372)	Ansamycin	*Micromonospora spp.*		2009	Antibacterial agent esp. active against	[158]

					mycobacteria.	
Butremycin(377)	Macrolactam	*Micromonospora sp. K310*	Marine	2014	Antibacterial	[159]
lobosamides A-C (378-380)	Macrolactam	Micromonospora sp.	Marine	2015	Antiparasitic activity mainly A IC_{50}(0.8 μM)	[160]
FW05328-1 (381)	Macrolactam (Ansamycin)	*Micromonospora* sp. FIM05328	Soil	2018	Antiproliferative IC_{50}(0.00020-30.77μM.	[161]
Aurodox (382)	Macrolactam (Ansamycin)	*Micromonospora* sp. FIM05328	Soil	2018	Antiproliferative IC_{50} (20.56-83.76 μM).	[161]
Microansamycins A−I (383-391)	Macrolactam (ansamycin)	*Micromonospora sp.*	--------	2018	Weak antioxidant only compound B with IC_{50} 0.85 mmol/L. Compounds A-C showed antibacterial activity with MICs 0.016- 0.5 μg/mL. Compounds D-F showed moderate antibacterial activity with MICS 1-8 μg/mL.	[162]
Sporalactam A (392)	Ansa Macrolide	*Micromonospora sp.*	marine sediment	2017	Antibacterial (0.8-7 μM)).	[163]
Sporalactam B (393)	Ansa Macrolide	*Micromonospora sp.*	marine sediment	2017	Antibacterial (0.06 -1.8 μM)).	[163]

3-amino-27-demethoxy-27-hydroxyrifamycin S (394)	Ansa Macrolide	*Micromonospora sp.*	marine sediment	2017	Antibacterial (0.0001-0.0009μM)	[163]
3-amino-rifamycin S (395)	Ansa Macrolide	*Micromonospora sp.*	marine sediment	2017	Antibacterial (0.0001-0.0008 μM)	[163]
Hazimicins (5 and 6) (396,397)	Nitriles	*M. echinospora* var. *challisensis* SCC 1411	Soil	1983	Antibacterial Antifungal	[164]
Antibiotic Y 03559J-A (398)	Isonitrile	*Micromonospora sp.* Y-03559J	Soil	1995	Antibacterial	Japan. Pat., 1995, 95 02 821.
YM-47515 (399)	Isonitrile compound	*M. echinospora* subsp. *echinospora* Y-03559J	Soil	1997	Antibacterial activity Against gram positive bacteria zone of inhibition (17-36mm)	[165]
Retymicin (400)	Xanthone	*Micromonospora sp.* Tü 6368	Soil	2005	Cytostatic activity < 1μg/ml.	[134]
MDN-0185 (401)	Polycyclic Xanthone	*Micromonospora sp.* CA-256353	--------------	2018	Antiplasmodial IC50 (9 Nm).	[166]
Mycinonic acid III (402)	Fatty acid	*M. griseorubida*	Soil	1991	Proposed biosynth. intermed. of mycinamycins	[167]
Epimycinonic acid I (403)	Fatty acid	*M. griseorubida*	Soil	1991	Proposed biosynth. intermed. of mycinamycins	[167]
Mycinonic acid I Mycinonic acid II. Mycinonic acid IV Decarboxymycinonic acid III (404-407)	Fatty acid	*M. griseorubida*	Soil	1991	Proposed biosynth. intermed. of mycinamycins	[167]

3,15-Dihydroxy-4,6,8,14-tetramethyl-5,9-dioxo-10,12-heptadecadienoic acid (408)	Fatty acid	*M. griseorubida*	Soil	1992	NA	[168]
Saquayamycin Z (409)	Saquayamycin	*Micromonospora sp*. Tü 6368	Soil	2005	Cytostatic activity < 1µg/ml.	[134]
Psicofuranine (410)	Nucleoside-type antibiotic	*M. echinospora*	Soil	1959	Antitumour and antibacterial	[169]
7-Deazainosine (411)	Nucleoside antibiotic.	*M. chalcea*	Soil	1970	Cytotoxic	Japan. Pat., 1970, 20 559.
5,6-Dihydro-5-azathymidine (412)	Nucleoside antibiotic	*M. melanogenes*	Soil	1975	Antibacterial Antiviral	US Pat., 1975, 3 907 643.
Dapiramicin A (413)	Ribonucleoside	*Micromonospora sp*. SF-1917	Soil	1983	Antifungal Effective against sheath blight in rice plants caused by Rhizoctonia solani, and against Colletotrichum lagenarium on cucumbers	[170]
Epidapiramicin A (414)	Ribonucleoside	*Micromonospora sp*.	Soil	1984	Shows activity against sheath blight in rice plants. Less effective than Dapiramicin A	[171]
Sch 40832 (415)	Thiostrepton	*M. carbonacea* var. africana ATCC 39149	Soil	1998	Antibacterial activity	[97]

Streptimidone Ao58A (416)	Glutarimide	*M. coerulea* Ao58	Sea-mud soil	1999	Antifungal activity MIC (3-10μg/ml).	172
Maklamicin (417)	Spirotetronate polyketide	*Micromonospora sp.*GMKU326	Root of a leguminous plant.	2011	Antibacterial MIC (0.2-13μg/ml)<br><br>Anticancer IC_{50} (17-34 μM)	173
Neomacquarimicin (418)	Carbocylic polyketide	*Micromonospora sp.* NPS2077	Marine sponge	2014	-----------------	174
MBJ-0003 (419)	Hydroxamate metabolite	*Micromonospora sp.* 29867	Shellfish, Marine	2014	Cytotoxic IC_{50} (11μM)	175
7-Acetyl-3, 6-dihydroxy-8-methyl-1-tetralone. GTRI 02. L-form (420)	------------------	Micromonospora sp. SA246	Soil	1998	Lipid peroxidation inhibitor IC_{50} 1.89 μg/ml.	176
Naphthalenepropanoic acid (421)	------------------	*Micromonospora* sp. HS-HM-036	Muddy sea sediments	2016	Anticancer IC_{50} (46.5 μg/ml). Antibacterial activity MICS (0.016 and 8 mg/ml).	177
Serine alkaline proteases (422)	Enzymes	M. chaiyaphumensis S103	Sfax solar saltern .	2017	Deproteinization of shrimp waste. Detergent	178
2- phenylacetic acid (423)	Aromatic acid	*Micromonospora sp.* (strain G044)	sponge	2017	-----------------------	101

Diacidene (424)	Polyene Dicarboxylic Acid	Micromonospora coxensis MTCC 8093	Marine	2012	----------------------	[180]
Antibiotic XK 206 (425)	--------------------	*Micromonospora sp*	Soil	1980	Weak antibacterial	Japan. Pat., 1980, 80 15 409.
Deoxydehydrochorismic acid 3-(1 Carboxyvinyloxy)benzoic acid (426)	Aromatic acid	*M. coxensis*	Marine	2012	NA	[180]
Glutamine scyllo-inositol transaminase (427)	Aminotransferase enzyme	*M. purpure*	Soil	1989	Catalyses the reaction of L-glutamine with 2,4,6/3,5-pentahydroxycyclohexanone to give 2-oxoglutaramate and 1-amino-1-deoxy-scyllo-inosito	[181]
Glyphomicin (428)	Phosphoglycolipid	*Micromonospora sp.* ATCC 53481	Soil	1989	Antibacterial	US Pat.,1989,4,842 857 [182]
M GCI (429)	Glycoprotein	*Micromonospora sp.* BR-1613	Soil	1984	β-Glucuronidase inhibitor	[183]
Isopimara-2-one-3-ol-8,15-diene(430)	Diterpene	*Micromonospora sp.*	Marine	2015	-------------	[117]
Micromonohalimanes A (431) and B (432)	Diterpene	Micromonospora sp.	Marine		Antibacterial	[184]
Daidzein-40-(2-deoxy-α-l-fucopyranoside)(433) Daidzein-7-(2-deoxy-α-l-fucopyranoside)(434) Daidzein-40,7-di-(2-	Isoflavonoid	*M. aurantiaca 110B*	Soil	2019	Moderate cytotoxic activity	[185]

deoxy-α-l-fucopyranoside)(435)						
Dimethyl phenazine-1,6-dicarboxylate (436) phenazine-1,6-dicarboxylic acid mono methyl ester(437) Phenazine-1-carboxylic acid; tubermycin(438)	Alkaloids	Micromonospora sp UR 56	Marine	2020	Antibacterial, antibiofilm and moderate cytotoxic activity	[186]
N-(2-hydroxyphenyl)-acetamide(439)	Aromatic acid	*Micromonospora* sp UR 56	Marine	2020	Cytotoxic	[186]
p-anisamide(440)	Aromatic acid	*Micromonospora* sp UR 56	Marine	2020	Antibacterial, antibiofilm and moderate cytotoxic activities	[186]
Paulomycin G (441)	*Paulomycin*	*M. matsumotoense M-412*	*Marine*	*2017*	*Antibacterial and cytotoxic activities*	[187]

References:

1 T. H. Haskell, J. C. French and Q. R. Bartz, *J. Am. Chem. Soc.*, 1959, **81**, 3480–3481.

2 G. M. Luedemann and B. C. Brodsky, *Antimicrob. Agents Chemother*, 1964, **1963**, 116–124.

3 Google Patents, US Patent, 3 ,454 696, 1969.

4 M. J. Weinstein, J. A. Marquez, R. T. Testa, G. H. Wagman, E. M. Oden and J. A. Waitz, *J. Antibiot. (Tokyo).*, 1970, **23**, 551–554.

5 J. Berdy, J. K. Pauncz, Z. M. Vajna, G. Horvath, J. Gyimesi and I. Koczka, *J. Antibiot. (Tokyo).*, 1977, **30**, 945–954.

6 G. H. Wagman, J. A. Marquez, P. D. Watkins, J. V Bailey, F. Gentile and M. J. Weinstein, *J. Antibiot. (Tokyo).*, 1973, **26**, 732–736.

7 G. H. Wagman, R. T. Testa, J. A. Marquez and M. J. Weinstein, *Antimicrob. Agents Chemother.*, 1974, **6**, 144–149.

8 B. K. Lee, R. G. Condon, G. H. Wagman and E. Katz, *Antimicrob. Agents Chemother.*, 1976, **9**, 151–159.

9 R. T. Testa, G. H. Wagman, P. J. L. Daniels and M. J. Weinstein, *J. Antibiot. (Tokyo).*, 1974, **27**, 917–921.

10 R. Okachi, I. Kawamoto, S. Takasawa, M. Yamamoto, S. Sato, T. Sato and T. Nara, *J. Antibiot. (Tokyo).*, 1974, **27**, 793–800.

11 M. J. Weinstein, G. H. Wagman, J. A. Marquez, R. T. Testa and J. A. Waitz, *Antimicrob. Agents Chemother.*, 1975, **7**, 246–249.

12 P. J. L. Daniels, C. Luce, T. L. Nagabhushan, R. S. Jaret, D. Schumacher, H. Reimann and J. a n Ilavsky, *J. Antibiot. (Tokyo).*, 1975, **28**, 35–41.

13 J. A. Marquez, G. H. Wagman, R. T. Testa, J. A. WALTZ and M. J. Weinstein, *J. Antibiot. (Tokyo).*, 1976, **29**, 483–487.

14 B. K. Lee, R. G. Condon, G. H. Wagman and M. J. Weinstein, *J. Antibiot. (Tokyo).*, 1976, **29**, 677–684.

15 M. Shimura, Y. Sekizawa, K. Iinuma, H. Naganawa and S. Kondo, *Agric. Biol. Chem.*, 1976, **40**, 611–618.

16 T. Nara, M. Yamamoto, I. Kawamoto, K. Takayama, R. y o Okachi, S. Takasawa, T. Sato and S. Sato, *J. Antibiot. (Tokyo).*, 1977, **30**, 533–540.

17 D. H. Davies, D. Greeves, A. K. Mallams, J. B. Morton and R. W. Tkach, *J. Chem. Soc. Perkin Trans. 1*, 1975, 814–818.

18 M. Kugelman, R. S. Jaret and S. Mittelman, *J. Antibiot. (Tokyo).*, 1978, **31**, 643–645.

19 M. Sugimoto, S. Ishii, R. Y. O. Okachi and T. Nara, *J. Antibiot. (Tokyo).*, 1979, **32**, 868–873.

20 H. Maehr, C.-M. Liu, T. Hermann, B. L. A. T. PROSSER, J. M. SMALLHEER and N. J. PALLERONI, *J. Antibiot. (Tokyo).*, 1980, **33**, 1431–1436.

21 Google Patents, United States patent US 4,187,299, 1980.

22 Google Patents, U.S. Patent 4,214,080, 1980.

23 Google Patents, US Pat., 4 214 080, 1980.

24 Google Patents, U.S. Patent 4,219,644., 1980.

25 J. R. Martin, P. Johnson, J. Tadanier and A. Goldstein, *Antimicrob. Agents Chemother.*, 1980, **18**, 761–765.

26 K. Shirahata, T. Iida, M. Sato and K. Mochida, *Carbohydr. Res.*, 1981, **92**, 168–175.

27 K. Shirahata, H. Kase, S. Kitamura and T. Iida, *J. Antibiot. (Tokyo).*, 1982, **35**, 520–523.

28 S. Kitamura, H. Kase, Y. Odakura, T. Iida, K. Shirahata and K. Nakayama, *J. Antibiot. (Tokyo).*, 1982, **35**, 94–97.

29 H. Kase, G. Shimura, T. Iida and K. Nakayama, *Agric. Biol. Chem.*, 1982, **46**, 515–522.

30 Y. Odakura, H. Kase, S. Iitoh, S. Satoh, S. Takasawa, K. Takahashi, K. Shirahata and K. Nakayama, *J. Antibiot. (Tokyo).*, 1984, **37**, 1670–1680.

31 S. Itoh, Y. Odakura, H. Kase, S. Satoh, K. Takahashi, T. Iida, K. Shirahata and K. Nakayama, *J. Antibiot. (Tokyo).*, 1984, **37**, 1664–1669.

32 J. P. Zhu, Y. F. Ni and L. Z. Xu, *Wei Sheng Wu Xue Bao*, 1987, **27**, 181–185.

33 M. a y d Lee, J. K. Manning, D. R. Williams, N. A. Kuck, R. T. Testa and D. B. Borders, *J. Antibiot. (Tokyo).*, 1989, **42**, 1070–1087.

34 M. Chu, R. Mierzwa, J. Jenkins, T.-M. Chan, P. Das, B. Pramanik, M. Patel and V. Gullo, *J. Nat. Prod.*, 2002, **65**, 1588–1593.

35 I. Schneemann, K. Nagel, I. Kajahn, A. Labes, J. Wiese and J. F. Imhoff, *Appl. Environ. Microbiol.*, 2010, **76**, 3702–3714.

36 T. Vályi-Nagy, J. Úri and I. Szilágyi, *Nature*, 1954, **174**, 1105.

37 J. Marquez, A. MURAWSKI, G. H. WAGMAN, R. S. JARET and H. REIMANN, *J. Antibiot. (Tokyo).*, 1969, **22**, 259–264.

38 J. A. Waitz, C. G. Drube, E. L. MOSS and M. J. WEINSTEIN, *J. Antibiot. (Tokyo).*, 1972, **25**, 647–652.

39 I. Kawamoto, R. Y. O. OKACHI, H. KATO, S. YAMAMOTO, I. TAKAHASHI, S. TAKASAWA and T. NARA, *J. Antibiot. (Tokyo).*, 1974, **27**, 493–501.

40 K. Hatano, E. Higashide and M. Shibata, *J. Antibiot. (Tokyo).*, 1976, **29**, 1163–1170.

41 T. Kishi, S. Harada, H. Yamana and A. Miyake, *J. Antibiot. (Tokyo).*, 1976, **29**, 1171–1181.

42 T. Furumai, I. Maezawa, N. Matsuzawa, S. Yano, T. Yamaguchi, K. Takeda and T. Okuda, *J. Antibiot. (Tokyo).*, 1977, **30**, 443–449.

43 T. Yamaguchi, H. Hayasaka, H. Yoshida, T. Matsushita, A. Yamabe and S. Ohshima, *J. Antibiot. (Tokyo).*, 1978, **31**, 433–440.

44 K. Kobinata, M. Uramoto, T. Mizuno and K. Isono, *J. Antibiot. (Tokyo).*, 1980, **33**, 772–775.

45 S. Satoi, N. Muto, M. Hayashi, T. Fujii and M. Otani, *J. Antibiot. (Tokyo).*, 1980, **33**, 364–376.

46 S. Omura, C. Kitao and H. Matsubara, *Chem. Pharm. Bull.*, 1980, **28**, 1963–1965.

47 R. W. Vaughan, J. Lotvin, M. S. Puar, M. Patel, A. Kershner, M. G. Kalyanpur, J. Marquez and J. A. Waitz, *J. Antibiot. (Tokyo).*, 1982, **35**, 251–253.

48 E. Martinelli, L. Faniuolo, G. Tuan, G. G. Gallo and B. Cavalleri, *J. Antibiot. (Tokyo).*, 1983, **36**, 1312–1322.

49 N. Sadakane, Y. Tanaka and S. Omura, *J. Antibiot. (Tokyo).*, 1983, **36**, 921–922.

50 T. Takatsu, H. Nakayama, A. Shimazu, K. Furihata, K. Ikeda, K. Furihata, H. Seto and N. Otake, *J. Antibiot. (Tokyo).*, 1985, **38**, 1806–1809.

51 H. Achenbach, A. Mühlenfeld, U. Fauth and H. Zähner, *Tetrahedron Lett.*, 1985, **26**, 6167–6170.

52 Y. Takahashi, Y. Iwai and S. Omura, *J. Antibiot. (Tokyo).*, 1986, **39**, 1413–1418.

53 H. Nakayama, T. Hanamura, Y. Abe, a Shimazu, K. Furihata, K. Ikeda, K. Furihata, H. Seto and N. Otake, *J. Antibiot. (Tokyo).*, 1986, **39**, 1016–1020.

54 B. Cavalleri, A. Arnone, E. Di Modugno, G. Nasini and B. P. Goldstein, *J. Antibiot. (Tokyo).*, 1988, **41**, 308–315.

55 H. Imai, K. Suzuki, M. Morioka, T. Sasaki, K. Tanaka, S. Kadota, M. Iwanami, T. Saito and H. Eiki, *J. Antibiot. (Tokyo).*, 1989, **42**, 1000–1002.

56 S. Nakajima, K. Kojiri, H. Morishima and M. Okanishi, *J. Antibiot. (Tokyo).*, 1990, **43**, 1006–1009.

57 M. S. Puar and D. Schumacher, *J. Antibiot. (Tokyo).*, 1990, **43**, 1497–1501.

58 K. Funaishi, K. Kawamura, F. Satoh, M. Hiramatsu, M. Hagiwara and M. Okanish, *J. Antibiot. (Tokyo).*, 1990, **43**, 938–947.

59 K. Kinoshita, S. Takenaka and M. Hayashi, MitsuoKinoshita, K., Takenaka, S., Suzuki, H., Morohoshi, T., & Hayashi, *J. Antibiot. (Tokyo).*, 1991, **44**, 1270–1273.

60 K. W. Shimotohno, T. Endo and K. Furihata, *J. Antibiot. (Tokyo).*, 1993, **46**, 682–684.

61 Y. Hayakawa, K. Shin-Ya, K. Furihata and H. Seto, *J. Antibiot. (Tokyo).*, 1993, **46**, 1563–1569.

62 K. Yasumuro, M. Shibazaki, T. Sasaki, H. Imai, H. Yamaguchi, K. Suzuki, M. Morioka and Y. Takebayasi, *J. Antibiot. (Tokyo).*, 1994, **47**, 250–252.

63 Z. Hepin and G. Juefen, *Pharm. Biotechnol.*, 1994, 1.

64 K. S. Lam, G. A. Hesler, D. R. Gustavson, R. L. Berry, K. Tomita, J. L. MacBeth, J. Ross, D. MiLLER and S. FORENZA, *J. Antibiot. (Tokyo).*, 1996, **49**, 860–864.

65 D. R. Schroeder, K. L. Colson, S. E. Klohr, M. S. Lee, J. A. Matson, L. S. Brinen and J. Clardy, *J. Antibiot. (Tokyo).*, 1996, **49**, 865–872.

66 G. H. Harris, A. Shafiee, M. A. Cabello, J. E. Curotto, O. Genilloud, K. E. Göklen, M. B. Kurtz, M. Rosenbach, P. M. Salmon and R. A. Thornton, *J. Antibiot. (Tokyo).*, 1998, **51**, 837–844.

67 L. M. Canedo, J. L. F. Puentes, J. P. Baz, X. H. Huang and K. L. Rinehart, *J. Antibiot. (Tokyo).*, 2000, **53**, 479–483.

68 V. R. Hegde, M. S. Puar, P. Dai, M. Patel, V. P. Gullo, P. R. Das, R. W. Bond and A. T. McPhail, *Tetrahedron Lett.*, 2000, **41**, 1351–1354.

69 E. Ohta, N. K. Kubota, S. Ohta, M. Suzuki, T. Ogawa, A. Yamasaki and S. Ikegami, *Tetrahedron*, 2001, **57**, 8463–8467.

70 J. A. Laakso, U. M. Mocek, J. Van Dun, W. Wouters and M. Janicot, *J. Antibiot. (Tokyo).*, 2003, **56**, 909–916.

71 C. Thawai, P. Kittakoop, S. Tanasupawat, K. Suwanborirux, K. Sriklung and Y. Thebtaranonth, *Chem. Biodivers.*, 2004, **1**, 640–645.

72 Y. Anzai, Y. Iizaka, W. Li, N. Idemoto, S. Tsukada, K. Koike, K. Kinoshita and F. Kato, *J. Ind. Microbiol. Biotechnol.*, 2009, **36**, 1013.

73 Y. Anzai, A. Sakai, W. Li, Y. Iizaka, K. Koike, K. Kinoshita and F. Kato, *J. Antibiot. (Tokyo).*, 2010, **63**, 325.

74 A. Gärtner, B. Ohlendorf, D. Schulz, H. Zinecker, J. Wiese and J. F. Imhoff, *Mar. Drugs*, 2011, **9**, 98–108.

75 S. Carlson, L. Marler, S.-J. Nam, B. Santarsiero, J. Pezzuto and B. Murphy, *Mar. Drugs*, 2013, **11**, 1152–1161.

76 P. Fei, W. Chuan-xi, X. Yang, J. Hong-lei, C. Lu-jie, P. Uribe, A. T. Bull, M. Goodfellow, J. Hong and L. Yun-yang, *Nat. Prod. Res.*, 2013, **27**, 1366–1371.

77 E. J. Skellam, A. K. Stewart, W. K. Strangman and J. L. C. Wright, *J. Antibiot. (Tokyo).*, 2013, **66**, 431.

78 M. C. Kim, H. Machado, K. H. Jang, L. Trzoss, P. R. Jensen and W. Fenical, *J. Am. Chem. Soc.*, 2018, **140**, 10775–10784.

79 K. Kobinata, M. Uramoto, T. Mizuno and K. Isono, *J. Antibiot. (Tokyo).*, 1980, **33**, 244–246.

80 F. Tomita, T. Tamaoki, K. Shirahata, M. Kasai, M. Morimoto, S. Ohkubo, K. Mineura and S. Ishii, *J. Antibiot. (Tokyo).*, 1980, **33**, 668–670.

81 T. Tamaoki, M. Kasai, K. Shirahata and F. Tomita, *J. Antibiot. (Tokyo).*, 1982, **35**, 979–984.

82 Y. Igarashi, K. Takagi, Y. Kan, K. Fujii, K.-I. HARADA, T. Furumai and T. Oki, *J. Antibiot. (Tokyo).*, 2000, **53**, 233–240.

83 C. Gui, S. Zhang, X. Zhu, W. Ding, H. Huang, Y.-C. Gu, Y. Duan and J. Ju, *J. Nat. Prod.*, 2017, **80**, 1594–1603.

84 W. P. Fisher, J. Charney and W. A. Bolhofer, *Antibiot. Chemother. (Northfield, Ill.)*, 1951, **1**, 571–572.

85 S. Nakamura, N. Tanaka and H. Umezawa, *J. Antibiot. (Tokyo).*, 1966, **19**, 10–12.

86 Google Patents, U.S. Patent 4,078,056, 1978.

87 H. Maehr, J. Smallheer, M. Chin, N. Palleroni, F. Weiss and C.-M. Liu, *J. Antibiot. (Tokyo).*, 1979, **32**, 531–532.

88 Y. Kawamura, Y. Yasuda and M. Mayama, *J. Antibiot. (Tokyo).*, 1981, **34**, 367–369.

89 J.-I. Shoji, H. Hinoo, T. Kato, K. Nakauchi, S. Matsuura, M. Mayama, Y. Yasuda and Y. Kawamura, *J. Antibiot. (Tokyo).*, 1981, **34**, 374–380.

90 Google Patents, U.S. Patent 4,692,333., 1987.

91 Google Patents, U.S. Patent 4,478,831., 1984.

92 T. Okazaki, R. Enokita, H. Miyaoka, T. Takatsu and A. Torikata, *J. Antibiot. (Tokyo).*, 1987, **40**, 917–923.

93 R. Cooper, A. N. N. C. Horan, F. Gentile, V. Gullo, D. Loebenberg, J. Marquez, M. Patel, M. S. Puar and I. Truumees, *J. Antibiot. (Tokyo).*, 1988, **41**, 13–19.

94 K. S. Lam, D. R. Gustavson, G. A. Hesler, T. T. Dabrah, J. A. Matson, R. L. Berry, W. C. Rose and S. Forenza, *J. Ind. Microbiol.*, 1995, **15**, 60–65.

95 K. D. McBrien, R. L. Berry, S. E. Lowe, K. M. Neddermann, I. Bursuker, S. Huang, S. E. Klohr and J. E. Leet, *J. Antibiot. (Tokyo).*, 1995, **48**, 1446–1452.

96 J. P. Baz, L. M. Canedo, J. L. F. Puentes and M. V. S. ELIPE, *J. Antibiot. (Tokyo).*, 1997, **50**, 738–741.

97 M. S. Puar, T. M. Chan, V. Hegde, M. Patel, P. Bartner, K. J. Ng, B. N. Pramanik and R. D. MacFarlane, *J. Antibiot. (Tokyo).*, 1998, **51**, 221–224.

98 A. K. Saksena, E. Jao, B. Murphy, D. Schumacher, T.-M. Chan, M. S. Puar, J. K. Jenkins, D. Maloney, M. Cordero and B. N. Pramanik, *Tetrahedron Lett.*, 1998, **39**, 8441–8444.

99 H. Lackner, I. Bahner, N. Shigematsu, L. K. Pannell and A. B. Mauger, *J. Nat. Prod.*, 2000, **63**, 352–356.

100 M. S. V. Gurovic, S. Müller, N. Domin, I. Seccareccia, S. Nietzsche, K. Martin and M. Nett, *Int. J. Syst. Evol. Microbiol.*, 2013, **63**, 3812–3817.

101 C. D. Tuan, D. T. M. Huong, T. B. Ngan, V. T. Quyen, M. Brian, C. Van Minh and P. Van Cuong, *Vietnam J. Sci. Technol.*, 2017, **55**, 251.

102 L. Chen, W. Zhao, H.-L. Jiang, J. Zhou, X.-M. Chen, Y.-Y. Lian, H. Jiang and F. Lin, *Tetrahedron*, 2018, **74**, 4151–4154.

103 W.-C. Liu, G. Astle, E. S. Wells Jr, W. H. Trejo, P. A. Principe, M. L. Rathnum, W. L. Parker, O. R. Kocy and R. B. Sykes, *J. Antibiot. (Tokyo).*, 1980, **33**, 1256–1261.

104 Y. Ikeda, S. Kondo, T. Sawa, M. Tsuchiya, D. Ikeda, M. Hamada, T. Takeuchi and H. Umezawa, *J. Antibiot. (Tokyo).*, 1981, **34**, 1628–1630.

105 T. Fehr, J.-J. Sanglier, W. Schuler, L. Gschwind, M. PONELLE, W. SCHILLING and C. WLOLAND, *J. Antibiot. (Tokyo).*, 1996, **49**, 230–233.

106 K. Tani, Y. ARAI and T. YAMAGUCHI, *J. Antibiot. (Tokyo).*, 1982, **35**, 1441–1447.

107 R. A. Nelson, J. A. Pope Jr, G. M. Luedemann, L. E. Mcdaniel and C. P. Schaffner, *J. Antibiot. (Tokyo).*, 1986, **39**, 335–344.

108 W. L. Russell, R. C. Pandey, C. P. Schaffner and H. M. Fales, *J. Antibiot. (Tokyo).*, 1988, **41**, 149–156.

109 W.-H. Yeo, B.-S. Yun, N.-I. Back, Y.-H. Kim, S.-S. Kim, E.-K. Park, K.-S. Whang and Y. u Seung-Hun, *J. Antibiot. (Tokyo).*, 1997, **50**, 546–550.

110 T. da S. Sousa, P. C. Jimenez, E. G. Ferreira, E. R. Silveira, R. Braz-Filho, O. D. L. Pessoa and L. V Costa-Lotufo, *J. Nat. Prod.*, 2012, **75**, 489–493.

111 Y. Matsuda, K. Asano, I. Kawamoto and H. Kase, *J. Antibiot. (Tokyo).*, 1987, **40**, 1092–1100.

112 G. U. Y. T. Carter, J. A. Nietsche, D. R. Williams and D. B. Borders, *J. Antibiot. (Tokyo).*, 1990, **43**, 504–512.

113 W.-H. Yeo, B.-S. Yun, Y.-S. Kim, S. H. Yu, H.-M. Kim, I.-D. Yoo and Y. H. Kim, *J. Antibiot. (Tokyo).*, 2002, **55**, 511–515.

114 H. Wang, S. L. Yeo, J. Xu, X. Xu, H. He, F. Ronca, A. E. Ting, Y. Wang, V. C. Yu and M. M. Sim, *J. Nat. Prod.*, 2002, **65**, 721–724.

115 T. Furumai, Y. Igarashi, H. Higuchi, N. Saito and T. Oki, *J. Antibiot. (Tokyo).*, 2002, **55**, 128–133.

116 W. Zhang, Z. Liu, S. Li, Y. Lu, Y. Chen, H. Zhang, G. Zhang, Y. Zhu, G. Zhang and W. Zhang, *J. Nat. Prod.*, 2012, **75**, 1937–1943.

117 M. W. Mullowney, E. HAinmhire, U. Tanouye, J. E. Burdette, C. Van Pham and B. T. Murphy, *Mar. Drugs*, 2015, **13**, 5815–5827.

118 A. Ismet, S. Vikineswary, S. Paramaswari, W. H. Wong, A. Ward, T. Seki, H. P. Fiedler and M. Goodfellow, *World J. Microbiol. Biotechnol.*, 2004, **20**, 523–528.

119 M. Konishi, H. Ohkuma, K. Matsumoto, T. Tsuno, H. Kamei, T. Miyaki, T. Oki, H. Kawaguchi, G. D. Vanduyne and J. O. N. Clardy, *J. Antibiot. (Tokyo).*, 1989, **42**, 1449–1452.

120 K. Shiomi, H. Iinuma, H. Naganawa, M. Hamada, S. Hattori, H. Nakamura, T. Takeuchi and Y. Iitaka, *J. Antibiot. (Tokyo).*, 1990, **43**, 1000–1005.

121 M. Konishi, H. Ohkuma, K. Matsumoto, K. Saitoh, T. Miyaki, T. Oki and H. Kawaguchi, *J. Antibiot. (Tokyo).*, 1991, **44**, 1300–1305.

122 M. Miyoshi-Saitoh, N. Morisaki, Y. Tokiwa, S. Iwasaki, M. Konishi, K. Saitoh and T. Oki, *J. Antibiot. (Tokyo).*, 1991, **44**, 1037–1044.

123 Y. Igarashi, M. E. Trujillo, E. Martínez-Molina, S. Yanase, S. Miyanaga, T. Obata, H. Sakurai, I. Saiki, T. Fujita and T. Furumai, *Bioorg. Med. Chem. Lett.*, 2007, **17**, 3702–3705.

124 C.-M. Xue, L. Tian, W.-H. Lin and Z.-W. Deng, *Nat. Prod. Res.*, 2009, **23**, 533–538.

125 Y. Igarashi, S. Yanase, K. Sugimoto, M. Enomoto, S. Miyanaga, M. E. Trujillo, I. Saiki and S. Kuwahara, *J. Nat. Prod.*, 2011, **74**, 862–865.

126 T. D. S. Sousa, P. C. Jimenez, E. G. Ferreira, E. R. Silveira, R. Braz-Filho, O. D. L. Pessoa and L. V. Costa-Lotufo, *J. Nat. Prod.*, 2012, **75**, 489–493.

127 K. Aiso, T. Arai, I. Shidara and K. Ogi, *J. Antibiot. (Tokyo).*, 1954, **7**, 1–6.

128 G. Cassinelli, F. Di Matteo, S. Forenza, M. C. Ripamonti, G. Rivola, F. Arcamone, A. Di Marco, A. M. Casazza, C. Soranzo and G. Pratesi, *J. Antibiot. (Tokyo).*, 1980, **33**, 1468–1473.

129 T. P. Tully, 1987, **48**, 652–653.

130 M. G. Nair, S. K. Mishra, A. R. Putnam and R. C. Pahdey, *J. Antibiot. (Tokyo).*, 1992, **45**, 1738–1745.

131 K. Ishigami, Y. Hayakawa and H. Seto, *J. Antibiot. (Tokyo).*, 1994, **47**, 1219–1225.

132 S.-W. Yang, T.-M. Chan, J. Terracciano, R. Patel, D. Loebenberg, G. Chen, M. Patel, V. Gullo, B. Pramanik and M. Chu, *J. Antibiot. (Tokyo).*, 2004, **57**, 601–604.

133 N. Adnani, M. G. Chevrette, S. N. Adibhatla, F. Zhang, Q. Yu, D. R. Braun, J. Nelson, S. W. Simpkins, B. R. McDonald and C. L. Myers, *ACS Chem. Biol.*, 2017, **12**, 3093–3102.

134 N. Antal, H.-P. Fiedler, E. Stackebrandt, W. Beil, K. Ströch and A. Zeeck, *J. Antibiot. (Tokyo).*, 2005, **58**, 95.

135 H. He, H. Y. Yang, S. W. Luckman, V. S. Bernan, G. Tsai, D. M. Roll and G. T. Carter, *Helv. Chim. Acta*, 2004, **87**, 1385–1391.

136 A. H. Banskota, M. Aouidate, D. Sørensen, A. Ibrahim, M. Piraee, E. Zazopoulos, A.-M. Alarco, H. Gourdeau, C. Mellon and C. M. Farnet, *J. Antibiot. (Tokyo).*, 2009, **62**, 565.

137 M. J. Weinstein, G. M. Luedemann, E. M. Oden and G. H. Wagman, *Antimicrob. Agents Chemother.*, 1964, **10**, 24–32.

138 D. R. Foster and M. J. Rybak, *Pharmacother. J. Hum. Pharmacol. Drug Ther.*, 1999, **19**, 1111–1117.

139 M. Chu, R. Mierzwa, M. Patel, J. Jenkins, P. Das, B. Pramanik and T.-M. Chan, *Tetrahedron Lett.*, 2000, **41**, 6689–6693.

140 O. Ando, H. Satake, K. Itoi, A. Sato, M. Nakajima, S. Takahashi, H. Haruyama, Y. Ohkuma, T. Kinoshita and R. Enokita, *J. Antibiot. (Tokyo).*, 1991, **44**, 1165–1168.

141 J. Itoh, H.-O. Watabe, S. Ishii, S. Gomi, M. Nagasawa, H. Yamamoto, T. Shomura, M. Sezaki and S. Konodo, *J. Antibiot. (Tokyo).*, 1988, **41**, 1281–1284.

142 Google Patents, United States patent 5,541,181, 1996.

143 L.-M. Yang, W. Rong-Yang, A. T. Mcphail, T. Yokoi and K.-H. Lee, *J. Antibiot. (Tokyo).*, 1988, **41**, 488–493.

144 W. M. Maiese, M. P. Lechevalier, H. A. Lechevalier, J. Korshalla, J. Goodman, M. J. Wildey, N. Kuck and M. Greenstein, *J. Antibiot. (Tokyo).*, 1989, **42**, 846–851.

145 O. Ando, M. Nakajima, K. Hamano, K. Itoi, S. Takahashi, Y. Takamatsu, A. Sato, R. Enokita, T. OKAZAKI and H. HARUYAMA, *J. Antibiot. (Tokyo).*, 1993, **46**, 1116–1125.

146 H. Reimann and R. Jaret, *Chem. Ind.*, 1967, **52**, 2173.

147 L. M. C. Hernandez, J. A. D. E. L. A. F. Blanco, J. P. Baz, J. L. F. Puentes, F. R. Millan, F. E. Vazquez, R. I. Fernandez-Chimeno and D. G. Gravalos, *J. Antibiot. (Tokyo).*, 2000, **53**, 895–902.

148 M.-T. Gutierrez-Lugo, G. M. Woldemichael, M. P. Singh, P. A. Suarez, W. M. Maiese, G. Montenegro and B. N. Timmermann, *Nat. Prod. Res.*, 2005, **19**, 645–652.

149 Z. Huang, J. Tang, H. Gao, Y. Li, K. Hong, J. Li and X. Yao, *Chin J Mar Drugs*, 2011, **30**, 29–33.

150 S. Nakanishi, S. Chiba, H. Yano, I. Kawamoto and Y. Matsuda, *J. Antibiot. (Tokyo).*, 1995, **48**, 948–951.

151 A. L. Stefanska, N. J. Coates, L. M. Mensah, A. J. Pope, S. J. Ready and S. R. Warr, *J. Antibiot. (Tokyo).*, 2000, **53**, 345–350.

152 H. He, W.-D. Ding, V. S. Bernan, A. D. Richardson, C. M. Ireland, M. Greenstein, G. A. Ellestad and G. T. Carter, *J. Am. Chem. Soc.*, 2001, **123**, 5362–5363.

153 S.-W. Yang, T.-M. Chan, J. Terracciano, D. Loenbenberg, G. Chen, M. Patel, V. Gullo, B. Pramanik and M. Chu, *J. Antibiot. (Tokyo).*, 2004, **57**, 345–347.

154 R. D. Charan, G. Schlingmann, J. Janso, V. Bernan, X. Feng and G. T. Carter, *J. Nat. Prod.*, 2004, **67**, 1431–1433.

155 M. J. Weinstein, G. M. Luedemann, E. M. Oden and G. H. Wagman, *Antimicrob. Agents Chemother.*, 1967, **7**, 435.

156 Google Patents, U.S. Patent 4,013,789., 1977.

157 A. K. Ganguly, Y. T. Liu, O. Z. Sarre and S. Szmulewicz, *J. Antibiot. (Tokyo).*, 1977, **30**, 625–627.

158 H. Huang, X. Wu, S. Yi, Z. Zhou, J. Zhu, Z. Fang, J. Yue and S. Bao, *Antonie Van Leeuwenhoek*, 2009, **95**, 143.

159 K. Kyeremeh, K. S. Acquah, A. Sazak, W. Houssen, J. Tabudravu, H. Deng and M. Jaspars, *Mar. Drugs*, 2014, **12**, 999–1012.

160 C. J. Schulze, M. S. Donia, J. L. Siqueira-Neto, D. Ray, J. A. Raskatov, R. E. Green, J. H. McKerrow, M. A. Fischbach and R. G. Linington, *ACS Chem. Biol.*, 2015, **10**, 2373–2381.

161 Y.-L. Nie, Y.-D. Wu, C.-X. Wang, R. Lin, Y. Xie, D.-S. Fang, H. Jiang and Y.-Y. Lian, *Nat. Prod. Res.*, 2018, **32**, 2133–2138.

162 J. Wang, W. Li, H. Wang and C. Lu, *Org. Lett.*, 2018, **20**, 1058–1061.

163 D. E. Williams, D. S. Dalisay, J. Chen, E. A. Polishchuck, B. O. Patrick, G. Narula, M. Ko, Y. Av-Gay, H. Li and N. Magarvey, *Org. Lett.*, 2017, **19**, 766–769.

164 J. A. Marquez, A. N. N. C. Horan, M. Kalyanpur, B. K. Lee, D. Loebenberg, G. H. Miller, M. Patel and J. A. Waitz, *J.*

Antibiot. (Tokyo)., 1983, **36**, 1101–1108.

165 T. Sugawara, A. Tanaka, H. Imai, K. Naga and K. Suzuki, *J. Antibiot. (Tokyo).*, 1997, **50**, 944–948.

166 F. Annang, I. Pérez-Victoria, G. Pérez-Moreno, E. Domingo, I. González, J. R. Tormo, J. Martín, L. M. Ruiz-Pérez, O. Genilloud and D. González-Pacanowska, *J. Nat. Prod.*, 2018, **81**, 1687–1691.

167 K. Kinoshita, S. Takenaka and M. Hayashi, *J. Chem. Soc. Perkin Trans. 1*, 1991, 2547–2553.

168 K. Kinoshita, S. Takenaka, H. Suzuki, T. Yamamoto, T. Morohoshi and M. Hayashi, *J. Chem. Soc. Chem. Commun.*, 1992, 957–959.

169 W. Schroeder and H. Hoeksema, *J. Am. Chem. Soc.*, 1959, **81**, 1767–1768.

170 T. Shomura, N. Nishizawa, M. Iwata, J. Yoshida, M. Ito, S. Amano, M. Koyama, M. Kojima and S. Inouye, *J. Antibiot. (Tokyo).*, 1983, **36**, 1300–1304.

171 N. Nishizawa, Y. Kondo, M. Koyama, S. Omoto, M. Iwata, T. Tsuruoka and S. Inouye, *J. Antibiot. (Tokyo).*, 1984, **37**, 1–5.

172 B. S. Kim, S. S. Moon and B. K. Hwang, *J. Agric. Food Chem.*, 1999, **47**, 3372–3380.

173 Y. Igarashi, H. Ogura, K. Furihata, N. Oku, C. Indananda and A. Thamchaipenet, *J. Nat. Prod.*, 2011, **74**, 670–674.

174 S. Sato, F. Iwata, T. Fukae and M. Katayama, *J. Antibiot. (Tokyo).*, 2014, **67**, 479.

175 T. Kawahara, M. Itoh, M. Izumikawa, I. Kozone, N. Sakata, T. Tsuchida and K. Shin-ya, *J. Antibiot. (Tokyo).*, 2014, **67**, 261.

176 W.-H. Yeo, B.-S. Yun, S.-S. Kim, E.-K. Park, Y.-H. Kim, I.-D. Yoo and S.-H. Yu, *J. Antibiot. (Tokyo).*, 1998, **51**, 952–953.

177 M.-Y. Gao, H. Qi, J.-S. Li, H. Zhang, J. Zhang, J.-D. Wang and W.-S. Xiang, *J. Asian Nat. Prod. Res.*, 2017, **19**, 930–934.

178 S. Mhamdi, N. Ktari, S. Hajji, M. Nasri and A. S. Kamoun, *Int. J. Biol. Macromol.*, 2017, **94**, 415–422.

179 M. Y. Gao, H. Qi, J. S. Li, H. Zhang, J. Zhang, J. D. Wang and W. S. Xiang, *J. Asian Nat. Prod. Res.*, 2017, **19**, 930–934.

180 B. Ohlendorf, D. Schulz, P. Beese, A. Erhard, R. Schmaljohann and J. F. Imhoff, *Zeitschrift für Naturforsch. C*, 2012, **67**, 445–450.

181 L. A. Lucher, Y.-M. Chen and J. B. Walker, *Antimicrob. Agents Chemother.*, 1989, **33**, 452–459.

182 Google Patents, U.S. Patent 4,842,857, 1989.

183 M. Uyeda, K. Suzuki and M. Shibata, *Agric. Biol. Chem.*, 1984, **48**, 29–35.

184 Y. Zhang, N. Adnani, D. R. Braun, G. A. Ellis, K. J. Barns, S. Parker-Nance, I. A. Guzei and T. S. Bugni, *J. Nat. Prod.*, 2016, **79**, 2968–2972.

185 R.-J. Wang, S.-Y. Zhang, Y.-H. Ye, Z. Yu, H. Qi, H. Zhang, Z.-L. Xue, J.-D. Wang and M. Wu, *Mar. Drugs*, 2019, **17**, 294.

186 M. S Hifnawy, H. M. Hassan, R. Mohammed, M. M Fouda, A. M. Sayed, A. A Hamed, S. F AbouZid, M. E. Rateb, H. A. Alhadrami and U. R. Abdelmohsen, *Mar. Drugs*, 2020, **18**, 243.

187 A. Sarmiento-Vizcaíno, A. F. Braña, I. Pérez-Victoria, J. Martín, N. De Pedro, M. D. la Cruz, C. Díaz, F. Vicente, J. L. Acuña and F. Reyes, *Mar. Drugs*, 2017, **15**, 271.
